# Exploring the advances in 2D materials as a quest for energy storage electrode materials

**DOI:** 10.1039/d5ra07292a

**Published:** 2026-02-24

**Authors:** Sebin Kariachan, Joshin Shibu, Prajitha Velayudhan, Sisanth Krishnageham Sidharthan, Pravitha Velayudhan, Sanu Mathew Simon, Hitoshi Kasai, Kohei Okubo, Sabu Thomas, Kouki Oka, Jibin Keloth Paduvilan

**Affiliations:** a International and Inter-University Centre for Nanoscience and Nanotechnology (IIUCNN), Mahatma Gandhi University Kottayam Kerala 686560 India sabuthomas@mgu.ac.in; b Rubber Technology Centre, Indian Institute of Technology Kharagpur 721302 Kharagpur India; c Govt. Polytechnic College Adoor Kerala 691551 India; d St. Teresa's College Ernakulum Kerala-682011 India; e Department of Physics, Mar Athanasius College Kothamangalam 686666 India; f Institute of Multidisciplinary Research for Advanced Materials, Tohoku University 2-1-1 Katahira, Aoba-ku Sendai Miyagi 980-8577 Japan oka@tohoku.ac.jp kohei.okubo.a7@tohoku.ac.jp; g School of Energy Materials, Mahatma Gandhi University Kottayam Kerala 686560 India; h Carbon Recycling Energy Research Center, Ibaraki University 4-12-1 Nakanarusawa Hitachi Ibaraki 316-8511 Japan; i Deuterium Science Research Unit, Center for the Promotion of Interdisciplinary Education and Research, Kyoto University Yoshida, Sakyo-ku Kyoto 606-8501 Japan; j Department of Chemistry, Mahatma Gandhi College Iritty Kannur Kerala 670703 India jibinkp999@gmail.com

## Abstract

The increasing demand for effective energy storage solutions has spurred research into innovative materials with exceptional properties. Among these, two-dimensional (2D) materials have become popular alternatives due to their unique mechanical, chemical, and electrical characteristics. This review highlights recent advances in using 2D materials-such as graphene, MXenes, and transition metal dichalcogenides (TMDs) for energy storage devices like batteries and supercapacitors. Graphene, known for its high conductivity and surface area, enhances charge storage when used as an anode. MXenes, a newer class with variable compositions, provide high capacitance for both anodes and cathodes. The large surface area and capacitance of graphene, along with the flexibility of MXenes, offer promising advantages. Additionally, combining 2D materials with carbon nanotubes and metal nanoparticles further improves storage performance. New hybrid architectures and composites that incorporate 2D materials optimise energy storage devices, increasing metrics such as energy density and cycling stability. Moreover, integrating 2D materials into flexible, wearable devices addresses the needs of portable electronics and IoT. The review links the structure, synthesis, and characterisation to the electrochemical behavior of each material and also captures recent advances in the last decade related to battery and supercapacitor applications. In addition, the review includes two tables that compare the performance of each type of material as well as an increased focus on emerging hybrid structures for next-generation energy storage systems.

## Abstract 2

2D materials such as graphene, MXenes, and transition metal dichalcogenides, through hybridization strategies, enable the realization of high-performance electrode architectures for next-generation battery and supercapacitor energy storage systems.

## Graphical abstract

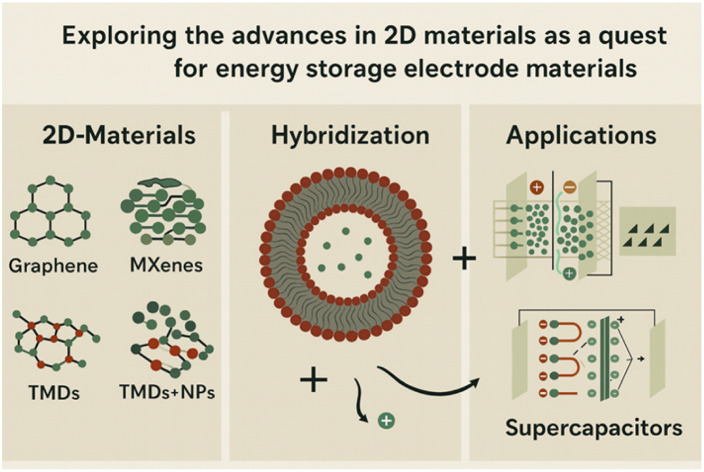

## 1. Introduction

The modern world is witnessing an unprecedented surge in the demand for efficient energy storage systems driven by the rapid growth of renewable energy sources, the electrification of transportation, and the increasing reliance on portable electronic devices. To facilitate the integration of renewable energy into the grid and to meet the rising energy demands, there is an urgent need for advanced energy storage technologies that can store and deliver energy effectively, reliably, and sustainably.^1^ Despite the remarkable progress in energy storage research, current technologies still face several limitations, such as low energy density, slow charging rates, limited cycle life, high cost, and environmental concerns^2^ associated with the use of conventional materials.

In response to these challenges, the exploration of novel materials with exceptional properties and tailored functionalities has become imperative.^3^ One class of materials that has emerged as a game-changer in the field of energy storage is two-dimensional (2D) materials. Unlike conventional bulk materials, 2D materials possess atomically thin structures, exhibiting unique electronic, mechanical, and chemical characteristics^4^ that make them promising candidates for addressing the limitations of current energy storage technologies. This review serves as an overview of the growing demand for efficient energy storage systems and the constraints faced by conventional technologies. Subsequently, we delve into the significance of 2D materials in mitigating these challenges, highlighting their exceptional properties and potential applications in various energy storage devices. Moreover, we will discuss recent advances in the utilization of 2D materials, focusing on batteries and supercapacitors, and outline the organization of this review paper.

### 1.1. Growing demand for efficient energy storage systems

The transition towards sustainable and low-carbon energy systems has led to a dramatic increase in the deployment of renewable energy sources, such as solar and wind power. While these renewable sources offer abundant energy, their intermittency poses a significant challenge to grid stability and reliability.^5^ Energy storage systems play a pivotal role in overcoming this intermittency, enabling efficient energy capture during surplus periods and discharge when demand peaks or renewable energy production is low.^6^ Additionally, the electrification of the transportation sector has also amplified the need for high-performance energy storage solutions to power electric vehicles (EVs) with extended driving ranges and reduced charging times.^7^

Furthermore, the exponential growth of portable electronic devices, ranging from smartphones to wearables, has heightened the demand for compact and long-lasting energy storage systems.^8^ The increasing reliance on these devices in daily life has accentuated the necessity for high-energy-density batteries that can sustain prolonged usage without frequent recharging.^6^ Thus, there is an urgent need to develop energy storage technologies that not only meet the rising energy demands but also address the environmental concerns associated with conventional fossil-fuel-based energy sources and materials.

### 1.2. Limitations of current energy storage technologies

Although several energy storage technologies exist today, each with its strengths and limitations, none are without compromise. Conventional lead-acid batteries, widely used in automotive applications, suffer from low energy density and limited cycle life.^9^ Lithium-ion batteries (LIBs), which dominate the portable electronics market and are increasingly being utilized in EVs, offer relatively higher energy density and longer cycle life.^7^ However, they still fall short of meeting the escalating energy storage demands for large-scale applications.

The high cost and limited availability of certain raw materials, especially those used in LIBs, such as cobalt, raise concerns about the long-term sustainability of current energy storage technologies.^10^ Additionally, the safety of LIBs, with their propensity for thermal runaway and fire hazards, necessitates the development of safer alternatives for energy storage.

On the other hand, supercapacitors, offer fast charging and discharging rates but generally have lower energy densities compared to batteries.^11^ The use of traditional carbon-based electrodes in supercapacitors limits their specific capacitance and energy storage capacity. As a result, there is a pressing need to explore novel materials and structures that can enhance the energy storage capabilities of supercapacitors.

### 1.3. Necessity of 2D materials in energy storage applications

Two-dimensional materials have emerged as a promising class of materials that could revolutionize the field of energy storage. These materials are composed of single or a few layers of atoms, resulting in their unique physicochemical properties and large surface area-to-volume ratios. One of the most well-known 2D materials is graphene, a single layer of carbon atoms arranged in a two-dimensional honeycomb lattice. Graphene exhibits exceptional electronic conductivity, mechanical strength, and thermal stability, making it a prime candidate for various energy storage applications.^12^

Apart from graphene, other 2D materials, such as transition metal dichalcogenides (TMDs), hexagonal boron nitride (h-BN), and MXenes, have also garnered significant attention for their potential in energy storage devices.^13^ TMDs offer a diverse range of electronic and electrochemical properties, making them suitable for both anode and cathode materials in batteries. h-BN, with its wide bandgap and excellent insulating properties, has been explored as a dielectric material in capacitive energy storage systems.^14^ MXenes, a family of 2D transition metal carbides and nitrides, exhibit tunable electronic properties and high surface area, making them attractive candidates for supercapacitors and batteries.^15^ However, challenges such as large-scale synthesis, cost-effectiveness, and long-term stability need to be addressed to unlock the full potential of 2D materials in commercial energy storage applications.

### 1.4. Scope

This review provides a consolidated and comparative overview of the major classes of two-dimensional (2D) materials that have emerged as promising electrode candidates for electrochemical energy storage, with a primary focus on graphene-based materials, MXenes, and transition metal dichalcogenides (TMDs). These material families are examined as representative platforms encompassing metallic, semiconducting, and surface-functionalized 2D systems, thereby enabling a balanced assessment of their structure–property relationships and electrochemical behaviour in batteries and supercapacitors. Particular attention is given to how atomic structure, surface chemistry, phase composition, and defect engineering govern ion transport, charge storage mechanisms, and cycling stability across different device configurations.

The review is structured to systematically link material structure to synthesis strategy to physicochemical properties to electrochemical performance, allowing direct comparison across the three 2D material families within a unified framework. Following an introduction to the fundamental characteristics and synthesis routes of each material class, their performance in lithium-ion, sodium-ion, and emerging multivalent batteries, as well as in electric double-layer and pseudocapacitive supercapacitors, is critically evaluated. Comparative tables are used to benchmark key performance metrics such as specific capacity, capacitance, rate capability, and cycling durability, highlighting both strengths and persistent limitations associated with each class.

In addition to established material systems, the review emphasizes emerging hybrid and composite architectures that integrate 2D materials with metal oxides, conductive polymers, carbon nanostructures, and molecular redox species to overcome challenges related to restacking, limited active sites, and long-term stability. Forward-looking perspectives are provided on strategies such as surface termination control, phase engineering, heterostructure design, and oxidation-resistant architectures, which are increasingly important for translating laboratory-scale performance into practical energy storage technologies. By combining comparative analysis with a materials-design perspective, this review aims to serve as a useful reference for guiding future development of robust, high-performance 2D-material-based electrodes. In addition to established 2D material systems, this review includes a dedicated forward-looking section that examines emerging electrode concepts such as single-atom catalysts on 2D supports, hydrogen-bonded polyoxometalate hybrids, and oxidation-resistant wide-band-gap architectures. This section critically positions these future materials within the broader landscape of energy storage research, emphasizing stability-driven design, hybridization strategies, and durability under high-voltage and long-term cycling conditions.

## 2. An outlook into the different 2D materials' properties and synthesis routes

### 2.1. Graphene

**Fig. 1:**
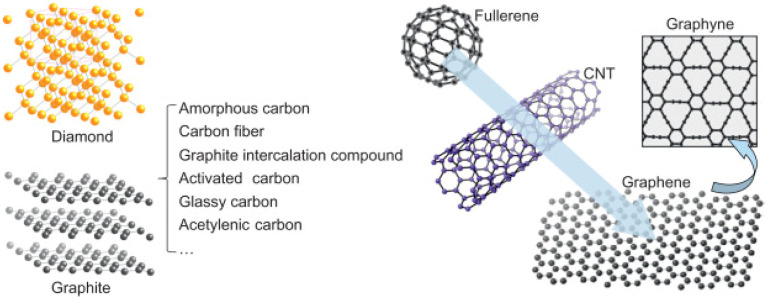
Carbon allotropes and related compounds reproduced from ref. 16 with permission from Elsevier, *Graphene*, Z. Zhen and H. Zhu, Elsevier, 2018, © 2018.

Graphene is a fascinating allotrope of carbon characterized by a monolayered distribution of carbon atoms arranged in a 2D layered honeycomb lattice structure. Each lattice of graphene is made up of three sigma bonds and exhibits sp^2^ hybridization, resulting in a robust hexagonal structure. The carbon–carbon bond length within a graphene layer is approximately 0.142 nm, while the overall thickness of the graphene is approximately 0.35 nm (Fig. 1).^16^

One of the most intriguing features of graphene is its exceptional electrical conductivity, primarily attributed to the presence of pi bonds perpendicular to the lattice plane. These pi bonds enable efficient electron mobility through the material, making graphene an outstanding conductor of electricity. This property is particularly beneficial in energy storage devices where fast electron transfer is important for performance. Additionally, the unique arrangement of carbon atoms in the hexagonal lattice confers remarkable mechanical strength and flexibility to the material, making it a promising candidate for various applications.^17^

When multiple layers of graphene are stacked together, they form other materials such as graphite, carbon nanotubes, fullerenes, and large aromatic molecules. Among these, few layer graphene and graphene-based composites are frequently utilised in batteries and supercapacitors to enhance conductivity, structural integrity, and ion diffusion pathways. Graphite, for instance, is composed of numerous graphene layers stacked on top of one another, with weak van der Waals forces holding the layers together. This arrangement allows the layers to slide over each other, giving graphite its characteristic lubricating properties.^18^ Carbon nanotubes are elongated cylindrical structures formed by rolling up a single layer of graphene.^19^ They possess extraordinary strength and unique electronic properties, making them valuable in a wide range of nanotechnology and materials science applications. Fullerenes, on the other hand, are spherical molecules consisting of carbon atoms arranged in a cage-like structure. Certain fullerenes, like buckminsterfullerene (C60), contain 60 carbon atoms, forming a geodesic dome-like shape.^20^ These fascinating molecules have captivated researchers due to their intriguing physical and chemical properties.

Graphene, with its sp^2^, hybridized carbon atoms and strong hexagonal lattice structure, lays the foundation for an array of remarkable carbon-based materials. Its exceptional electrical conductivity and mechanical properties, coupled with the ability to stack and form various structures, open up endless possibilities for applications in fields such as electronics, nanotechnology, and materials engineering.

#### 2.1.1. Properties of graphene

Graphene's excellent electrical conductivity, reaching around 10^6^ S m^−1^, makes it perfect for high-performance energy storage, enabling rapid charge and discharge in supercapacitors and batteries.^21^ Its outstanding mechanical strength, with a tensile strength near 125 GPa, maintains structural integrity in electrodes and prevents damage during repeated use. Moreover, graphene's very high in-plane thermal conductivity (∼5000 W m^−1^ K^−1^) helps dissipate heat effectively, enhancing thermal management and safety in battery systems.^7^ As a gapless semiconductor, its electronic properties can be precisely adjusted, making it suitable for hybrid energy devices. Additionally, graphene's impermeability to gases improves its role as a protective barrier in encapsulation layers and separators, boosting the lifespan and stability of energy storage systems. These qualities make graphene a key material for next-generation energy storage technologies.

• Optical transparency: due to its monolayered atomic structure and minimal thickness, graphene exhibits high optical transparency across a wide range of wavelengths.^21^ This property makes it an attractive candidate for transparent conductive films used in electronic devices, displays, and solar cells.

• Electrical conductivity: graphene stands out as the most conductive material known at room temperature, boasting a remarkable electrical conductivity of 106 S m^−1^ and a low sheet resistance of 31 Ω sq^−1^.^22^ This outstanding conductivity makes it highly suitable for use in electrical and electronic applications.

• Mechanical strength: graphene showcases extraordinary mechanical strength, making it the toughest crystal structure among all known materials. With a tensile strength of 125 GPa and an elastic modulus of 1.1 TPa,^23^ graphene exhibits exceptional resilience and robustness.

• Thermal conductivity: graphene's high in-plane thermal conductivity results from the strong covalent sp^2^ bonding between carbon atoms, enabling efficient heat transfer within the material.^24^ However, out-of-plane heat flow is restricted due to weak van der Waals coupling between graphene layers.

• Gapless semiconductor: one of the unique electronic properties of graphene arises from its gapless semiconductor nature. Charge carriers in graphene follow a linear dispersion relation, resembling massless relativistic particles.^25^ This leads to several intriguing electronic phenomena, contributing to their potential in electronic and optoelectronic devices.

• Transparent conductive membrane: the combination of optical transparency and electrical conductivity makes graphene an excellent candidate for transparent conductive membranes, used in applications like flexible touchscreens and wearable electronics.^26^

• High strength limit: graphene's impressive strength limit, reaching 42 N m^−1^, ensures its stability and resilience even under extreme conditions, making it suitable for various structural applications^27^.

• Gas barrier properties: single layers of graphene have shown excellent gas barrier properties, making it useful in applications where gas permeation needs to be controlled,^28^ such as in packaging and environmental protection.

#### 2.1.2. Different synthesis methods of graphene

Graphene can be synthesized through various methods, including both top-down and bottom-up approaches (Table 1):

**Table 1:** Different synthesis methods for graphene

Approach	Method	Principle/process	Advantages	Limitations
Top-down synthesis involves the production of graphene sheets through exfoliation or separation from highly ordered pyrolytic graphite (HOPG) or graphene oxide (GO)	Mechanical exfoliation (Scotch Tape method)	Applying stress to layered graphite using adhesive tape to isolate graphene layers.^29^	Produces high-quality, defect-free graphene; simple and low-cost	Very low yield; not scalable
	Chemical exfoliation	Formation of graphene-intercalated compounds, followed by exfoliation to yield graphene flakes.^30^	Can produce large quantities; relatively inexpensive	Flakes are small; presence of defects and functional groups
	Chemical synthesis (from graphene oxide, *etc.*)	Reduction of GO or precursor molecules to graphene sheets	Scalable; cost-effective	Introduces oxygen functional groups; reduced quality compared to pristine graphene
Bottom-up involves building up nanoscale materials by atomic or molecular arrangement of carbon to create graphene sheets.^31^	Pyrolysis (from precursors like sodium ethoxide)	Sonication detaches graphene sheets from pyrolyzed precursors.^32^	Produces high-quality graphene sheets	Limited scalability; costly precursors
	Epitaxial growth on SiC	Heating SiC substrate to sublime silicon, leaving behind graphene layers.^33^	Produces large-area, single-crystal graphene; excellent structural integrity	Very expensive; requires SiC substrates
	Chemical vapor deposition (CVD)	Hydrocarbon gases (*e.g.*, methane) decompose at high temperature, depositing graphene on metal substrates (*e.g.*, Cu, Ni).^34^	Produces large-area, high-quality graphene; scalable	Requires transfer process; potential defects introduced during transfer
	Substrate-free gas synthesis (microwave plasma reactor)	Graphene formed directly in gas phase without substrate.^35^	Substrate-independent; rapid synthesis	Quality control issues; less established method

Graphene has shown impressive electrochemistry in many forms of energy storage technology. Hybrid graphene/MnO_2_ electrodes showed a capacitance of 350 F g^−1^ at a current density of 1 A g^−1^ with 92% cycling retention, which can be indicative of their ability to function well as high power supercapacitors.^36^ The same was true for nitrogen-doped graphene, which produced a high reversible capacity of 875 mAh g^−1^ at a low rate of 0.1 A g^−1^ as an anode in a lithium-ion battery due to its increased conductivity, as well as its defect induced active sites.^37^ Additionally, three-dimensional (3D) porous graphene aerogels created a high energy density of 22 Wh kg^−1^ in symmetric supercapacitors by allowing for better ion transport pathways as well as providing greater structural integrity.^38^

### 2.2. Graphene oxide (GO)

Graphene Oxide (GO) is a unique monolayer material composed of carbon, hydrogen, and oxygen molecules.^39^ Its sp^2^ hybridization is disrupted, leading to properties that align more with those of an insulator rather than a conductor. Despite its insulating behavior, GO exhibits high hydrophilicity and can form stable aqueous colloids.^40^

This adjustable chemistry makes GO a promising precursor for energy storage uses. When partially reduced to reduced graphene oxide (rGO), it restores electrical conductivity while maintaining oxygen groups, enabling it to act as a conductive and chemically active matrix for battery electrodes and supercapacitors materials.

Several structural models have been proposed to describe the composition and arrangement of functional groups in GO:

➢ Hoffman and Holst's model: they suggested a structure with epoxy groups dispersed across the basal planes of graphite, leading to a net molecular formula of C2O.

➢ Reuss' variation:^41^ Reuss incorporated hydroxyl groups into the basal plane, accounting for the hydrogen content in GO. Additionally, the basal plane structure was altered to a sp^3^ hybridized system.

➢ Scholz and Boehm's model:^42^ this model removed the epoxide and ether groups completely and introduced regular quinoidal species, resulting in a corrugated backbone.

➢ Nakajima and Matsuo's assumption:^43^ They relied on the assumption of a lattice framework similar to poly(dicarbon monofluoride), a stage 2 graphite intercalation compound (GIC).

**Fig. 2:**
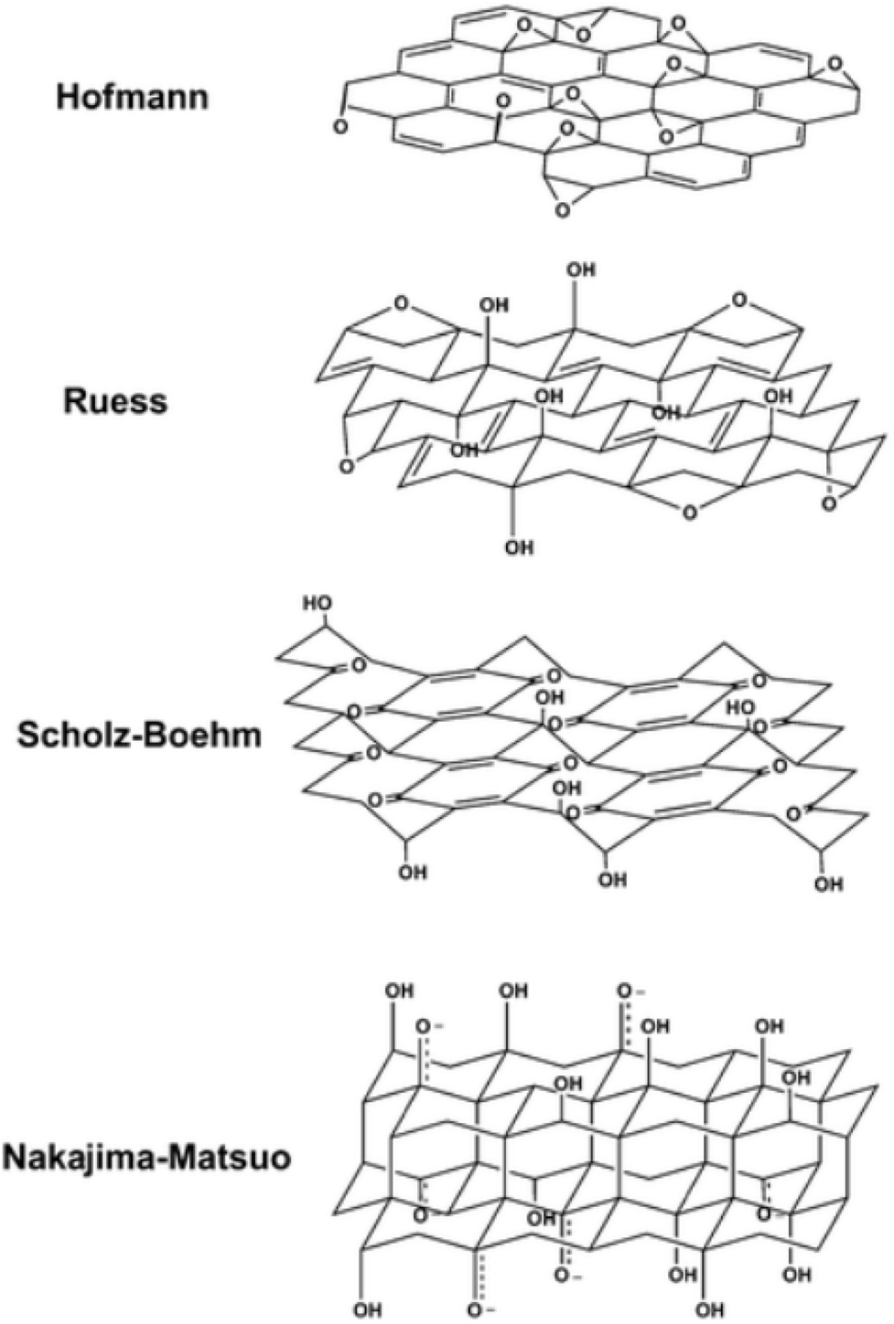
Different structures of graphene oxide, reproduced from ref. 44 with permission from the American Chemical Society, *Chemistry of Materials*, T. Szabó, O. Berkesi, P. Forgó, *et al.*, 2006, © 2006 American Chemical Society.

The various models proposed reflect the complexity and diversity of functional groups and hybridization patterns present in GO. The structural understanding of GO is crucial for tailoring its properties and optimizing its applications in areas such as energy storage, sensing, and biomedical devices. Despite its insulating nature, GO's unique hydrophilicity and colloidal stability make it a versatile material for various applications, including water purification, drug delivery, and as a precursor for the synthesis of reduced graphene oxide (rGO), which exhibits enhanced electrical conductivity compared to GO (Fig. 2).

#### 2.2.1. Properties of graphene oxide (GO)

• Mechanical properties: monolayer graphene oxide exhibits a lower effective Young's modulus (207.6 ± 23.4 GPa at 0.7 nm thickness) than pristine graphene. GO membranes made *via* solution-based deposition have a lower prestress (39.7–76.8 MPa) than mechanically cleaved graphene, indicating reduced mechanical strength and flexibility.^31^

• Electrical properties: GO's electrical resistance can be reduced through thermal annealing, chemical reduction, or the use of reducing agents.^45^ These processes restore the sp^2^ bonding orbitals and remove surface functional groups, increasing electrical conductivity. This improvement in electrical properties enhances GO's performance in electronic applications.

• Thermal properties: GO synthesized from graphite exhibits a low thermal conductivity of 0.5^−1^ W m^−1^ K^−1^.^46^ While high thermal conductivity is desirable for many applications, GO's low thermal conductivity can be advantageous in specific situations. For example, it can be used to provide high thermal insulation in applications such as home insulation and flame retardants. GO has been shown to be an effective filler in polymer nanocomposites to improve their flame-retardant properties.^47^

GO possesses oxygen functional groups that enhance its dispersion in water and organic solvents, facilitating easy integration with active materials like metal oxides and conductive polymers.^48,49^ These groups also promote strong interfacial interactions, improving the stability and performance of composites. Although GO is initially insulating, a controlled reduction converts it into reduced graphene oxide (rGO), which has tunable electrical properties—important for boosting electrode efficiency in energy storage devices. Its high surface area provides abundant active sites for ion adsorption in supercapacitors and improves electrode–electrolyte contact in batteries. Additionally, their mechanical flexibility makes them ideal for next-generation flexible and wearable energy devices, where durability and adaptability are essential.^50^

GO and reduced graphene oxide (rGO) composites are essential for enhancing energy storage device performance. In supercapacitors, combining GO/rGO with pseudocapacitive materials like MnO_2_, Ni(OH)_2_, or conductive polymers such as polyaniline and polypyrrole significantly boosts charge storage and energy density through synergistic effects.^51^ For lithium and sodium-ion batteries, GO acts as a stabilizing backbone for silicon and metal oxide anodes, minimising damaging volume expansion during cycling. It also serves as a conductive scaffold in sulfur-based cathodes, improving charge transfer in Li–S and Na–S batteries.^52^ In emerging zinc-ion and aluminum-ion systems, GO/rGO promotes faster ion diffusion and prevents metal dendrite formation at electrodes. Recent advances demonstrate these composites' promise, with GO-enhanced Li-ion anodes achieving capacities over 1000 mAh g^−1^ and supercapacitor electrodes surpassing 300 F g^−1^, all with excellent long-term cycling stability, making them promising for next-generation energy storage.^53,54^

#### 2.2.2. Different synthesis methods of graphene oxide

The synthesis of graphene oxide (GO) can be categorized into two main methods: bottom-up and top-down approaches. Bottom-up methods involve constructing pristine graphene from simple carbon molecules, while top-down methods extract graphene derivatives from a carbon source, typically graphite.

Bottom-up synthesis: techniques like chemical vapor deposition (CVD)^55^ and epitaxial growth on silicon carbide wafers are considered bottom-up approaches for GO synthesis. However, these methods have been found to be time-consuming and face challenges in scalability.

**Fig. 3:**
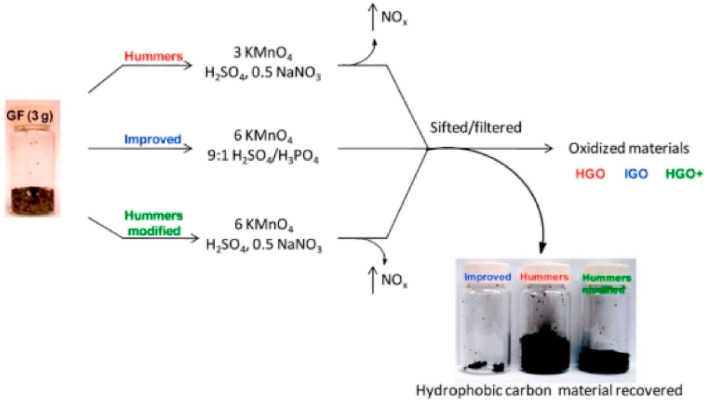
Schematic of the most common GO synthesis methods reproduced from ref. 59 with permission from the American Chemical Society, *ACS Nano*, D. C. Marcano, D. V. Kosynkin, J. M. Berlin, *et al.*, 2010, © 2010 American Chemical Society.

Top-down synthesis: the first synthesis of GO is credited to Brodie, Staudenmaier,^56^ and Hummers and Offeman. Hummers and Offeman's method,^57^ in particular, became widely used due to its safer and more scalable nature. It involves the oxidation of graphite using KMnO_4_ as the oxidizer, followed by the addition of sodium nitrate to form nitric acid *in situ*. The resulting Hummers' method, or slightly modified versions of it, are commonly employed for generating GO (Fig. 3).^58^

In the Hummers' method, a carbon source (typically graphite flakes or powders) is placed in a protonated solvent (*e.g.*, sulfuric acid or phosphoric acid) with the introduction of a strong oxidizing agent (usually KMnO_4_). The mixture is then diluted, treated with H_2_O_2_ to remove metal ions from the oxidizer, resulting in a yellow–brown liquid. The solids are separated, treated with dilute hydrochloric acid to further remove any metal species, and then washed and centrifuged with water until the solution's pH is nearly neutral.

Novel approaches to GO synthesis have been proposed, such as stationary oxidation mechanism,^60^ which leads to larger GO sheets. Another popular modification is the “improved Hummers” method,^61^ which omits sodium nitride and incorporates phosphoric acid and a higher amount of KMnO_4_. This modification eliminates toxic gases, enables easy temperature control, and produces GO powders with a higher degree of oxidation. The diverse methods of GO synthesis allow researchers to tailor the material's properties for specific applications, from electronics and energy storage to biomedical devices and composites.

As well as showing good energy storage properties, rGO and GO are promising materials in composite and hybrid forms. rGO/Fe_3_O_4_ composite nanomaterials showed high capacities (1120 mAh g^−1^) at low rates (0.1 A g^−1^), when tested as anode materials in lithium-ion batteries, thanks to both redox reactions and conductivity provided by the rGO network;^62^ similarly, GO hydrogel electrodes showed 210 F g^−1^ specific capacitance in aqueous supercapacitor systems because of their open pore structure and connectivity;^63^ Finally, rGO/polyaniline film electrodes displayed long term cycling capabilities, retaining more than 85% of their capacitance through 10 000 cycles, and thus could be considered for use in flexible or wearable devices.^64^

### 2.3. Reduced graphene oxide

Graphene oxide (GO) is a form of graphene that contains oxygen functional groups. When GO is subjected to reduction, these functional groups are eliminated, resulting in the formation of reduced graphene oxide (rGO). Although rGO still possesses some oxide functionalities, its structure becomes more similar to graphite, with much wider interlayer spacing (ranging from 6 to 12 Å) due to the insertion of water molecules.^65,66^

**Fig. 4:**
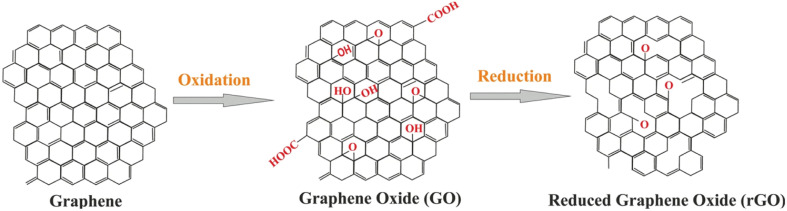
Reduction of GO to rGO reproduced from ref. 67 N. Balqis, B. M. Jan, H. S. C. Metselaar, A. Sidek, G. Kenanakis and R. Ikram, *Materials*, 2023, **16**, 3726, © 2023. The authors (CC BY 4.0).

The reduction process of GO to rGO involves the removal of oxygenated functional groups, leading to a material that retains the basic graphene framework but with improved electrical conductivity and other desirable properties. The wider interlayer spacing in rGO allows for enhanced interactions with various molecules and ions, making it a promising material for various applications, including energy storage, sensors, and catalysis. The unique combination of graphene-like structure and residual oxide functionalities in rGO makes it a versatile and valuable material for emerging technologies (Fig. 4).

The removal of oxygen functional groups during reduction leads to the restoration of sp^2^ hybridization in the graphene lattice, resulting in improved electrical conductivity compared to GO. This enhanced conductivity makes rGO a promising candidate for applications in electronics, such as flexible and transparent conductive films and electrodes for energy storage devices.

Furthermore, the wider interlayer spacing in rGO allows for greater accessibility and diffusion of molecules and ions, making it advantageous for applications in gas sensing, water purification, and electrochemical catalysis. The residual oxide functionalities in rGO also provide sites for chemical functionalization, enabling tailored surface modifications and improved compatibility with other materials.

#### 2.3.1. Properties of reduced graphene oxide (rGO)

• Mechanical properties: Gomez-Navarro^68^ reported that an rGO monolayer, produced by the original Hummers' method followed by thermal annealing in a hydrogen environment, exhibited a Young's modulus of 250 ± 150 TPa. The reduced GO sheets also demonstrated remarkable stability in their electrical properties against deformation. Electrical resistance measurements before and after repeated deformation showed that the resistance of the monolayers remained unchanged as long as the indentation depth was less than 10 nm. This mechanical and electrical stability makes rGO an attractive material for applications where consistent and reliable electrical contacts are required during deformation.^69^

• Electrical properties: reduction of GO improves its electrical conductivity. However, the presence of residual sp^3^-bonded carbon to oxygen in rGO restricts the movement of charge carriers through the sp^2^ clusters. As a result, the electrical transport in rGO occurs mainly through hopping, which is different from mechanically exfoliated graphene. Despite this, the improved electrical properties of rGO make it a promising conductive filler in polymer matrices for various applications, especially in electronic devices.^70,71^

• Thermal properties: rGO exhibits enhanced thermal conductivity, primarily due to its high conductivity, providing a low-resistance path for phonons owing to the large aspect ratio and interfacial contact area. Annealing GO at high temperatures (1000 °C) to produce rGO films can significantly improve in-plane thermal conductivity, increasing it from 3 to 61 W (m^−1^ K^−1^). These films also display anisotropic thermal conductivity, with a notable cross-plane thermal conductivity of 0.09 W (m^−1^ K^−1^) and an in-plane to cross-plane thermal conductivity ratio of 675. This anisotropy can be advantageous for applications that require directional heat conduction.^72^

rGO provides several key advantages for energy storage thanks to its unique structural and electronic properties. By restoring part of its conjugated carbon network, it significantly boosts electrical conductivity compared to GO, facilitating efficient charge transfer in battery electrodes and supercapacitor films. Its larger interlayer spacing (∼6–12 Å) enables rapid ion intercalation, which is particularly beneficial for multivalent batteries like zinc-ion and aluminum-ion types, where larger ions must move freely. The material also maintains mechanical flexibility and stability, ensuring long-lasting performance in flexible and wearable energy storage devices.^70,71^ Additionally, the remaining oxygen groups on rGO act as anchoring sites for nanoparticles, metal ions, and polymers, allowing for the creation of tailored composites that improve electrochemical performance.

rGO plays a key role in improving lithium-ion and sodium-ion batteries by forming a conductive network around high-capacity electrode materials like Sn, Si, and Fe_3_O_4_. Its flexible yet strong structure helps buffer the significant volume changes these materials undergo during charge and discharge cycles, boosting the electrode's stability and lifespan. In some cases, rGO acts as an anode material itself, offering reversible capacities ranging from 300 to 600 mAhg^−1^. Its performance is influenced by factors such as porosity and the number of layers stacked.^73,74^

In supercapacitor applications, rGO enhances both electric double-layer capacitance (EDLC) and pseudocapacitance when paired with metal oxides or conductive polymers. This dual role allows for specific capacitances between 200 and 350 F g^−1^, while also providing excellent rate capability, which is essential for high-power uses.^51^

Beyond conventional energy storage devices, rGO functions as a flexible conductive framework in hybrid systems. It forms the foundation for advanced ternary composites like rGO/metal oxide/polymer and acts as lightweight current collectors in flexible electronics, highlighting its adaptability for future energy storage technologies. These diverse roles make rGO an essential element in modern electrochemical energy storage systems.

#### 2.3.2. Different synthesis methods of reduced graphene oxide

Synthesis of reduced graphene oxide (rGO) involves the removal of oxygen functional groups from Graphene Oxide (GO) through thermal, chemical, or electrochemical methods. Several key factors must be considered during GO reduction, including achieving the desired C/O ratio, selectively removing oxygen groups, repairing surface defects, and preserving the physical and chemical properties of GO, such as mechanical strength, conductivity, optical properties, and solubility/dispersibility of nanosheets.^75,76^

Thermal reduction: GO can be thermally reduced through annealing in an oxygen-free environment or unconventional methods like microwaving GO powder or flash reduction of GO films using high-intensity light. These approaches offer simplicity and scalability but require careful control to avoid over-reduction and preserve the desired properties of rGO.^76^

Chemical reduction: chemical reducing agents are commonly added to GO solutions for reduction, and several well-supported techniques are available in the literature. These agents can include hydrazine, metal hydrides, hydrohalic acids, and other suitable chemicals. Careful selection and control of the reducing agent are essential to achieve the desired reduction level while minimizing unwanted side reactions.^77^

Photocatalyzed reduction: rGO can also be synthesized through photocatalyzed reactions. UV light in the presence of a TiO_2_ catalyst has been shown to effectively reduce GO, as demonstrated by the previous study.^78^ This approach provides a green and energy-efficient alternative to traditional reduction methods.

Electrochemical reduction: electrochemical reduction of GO is a chemical-agent-free process that relies on electron exchange between GO and the electrodes of an electrochemical cell. This approach offers precise control over the reduction process and has been explored as a promising method for producing rGO with tailored properties.^79^

In recent years, there has been growing interest in “green” reducing agents for synthesizing rGO, such as ascorbic acid, sugars, amino acids, and even microorganisms. These green methods offer environmentally friendly alternatives and reduce the generation of chemical waste. The choice of reduction method depends on the specific requirements of the application, scalability, energy usage, and waste management considerations. Researchers are continually exploring and optimizing various reduction strategies to tailor the properties of rGO for diverse applications, including electronics, energy storage, sensors, and composites.

### 2.4. MXenes

MXenes are a family of transition metal carbides, nitrides, or carbonitrides that are derived from the chemical delamination of 3D ternary (or quaternary) compounds known as MAX phases. These MAX phases serve as precursors to MXenes and have the general stoichiometry M_*n*+1_AX_*n*_, with *n* = 1, 2, or 3. Here, “M” represents a d-block transition metal, “A” denotes group 13 and 14 elements like Si, Al, Ge, or Sn, and “X” consists of carbon, nitrogen, or a combination of both.^80^

The structure of MAX phases features a hexagonal arrangement with space group *P*6_3_/*mmc*, where layers of “M” and “A” elements are interleaved, and “X” atoms occupy the octahedral sites formed by “M” elements. This layered structure allows for the exfoliation of MAX phases into 2D MXenes by selectively removing the “A” layer, leaving behind the transition metal carbide or nitride layers.^81,82^

MXenes are denoted by the general formula M_*n*+1_X_*n*_T_*x*_ (*n* = 1–3), where “M” represents various transition metals like Sc, Ti, Zr, Nb, and others, “X” represents carbon and/or nitrogen, and “T_*x*_” accounts for the hydroxyl, oxygen, or fluorine terminations that result from the synthesis procedures.^82^

In addition to being derived from MAX phases, MXenes can also be obtained from other layered compounds such as Zr_3_Al_3_C_5_ and Mo_2_Ga_2_C. With more than 70 known MAX phases and new discoveries being made regularly, MXenes present a rich and diverse family of 2D materials with fascinating properties and potential applications in various fields, including energy storage, catalysis, and electronics.^83^

The unique properties of MXenes, stemming from their 2D layered structure and tunable surface terminations, make them highly versatile and promising materials for a wide range of applications. Due to their excellent electrical conductivity, MXenes have found applications in energy storage devices, such as supercapacitors and lithium-ion batteries, where they demonstrate superior performance compared to conventional materials. Their high surface area, tunable surface chemistry, and catalytic activity have also led to their use as efficient catalysts in various chemical reactions.^84^

Moreover, MXenes exhibit exceptional mechanical strength and flexibility, making them attractive candidates for use in flexible electronics and wearable devices. Their tunable electronic band structures, ranging from metallic behavior to semiconducting properties, offer opportunities for designing novel electronic and optoelectronic devices. Additionally, the large interlayer spacing in MXenes enables facile intercalation of different ions and molecules, making them promising candidates for ion sieving membranes, sensors, and gas separation applications.

#### 2.4.1. Properties of MXene

MXenes are a revolutionary class of materials for energy storage, showcasing exceptional versatility in various device designs. In supercapacitors, they uniquely blend electric double-layer capacitance with redox-based pseudocapacitance, reaching industry-leading volumetric capacitance over 1500 F cm^−3^ and specific capacitance between 400–700 F g^−1^. Their inherent flexibility, ease of processing in solutions, and compatibility with aqueous electrolytes make them ideal for next-generation wearable devices and micro-supercapacitors integrated on chip.^83^

When used as anodes in lithium-ion and sodium-ion batteries, MXenes provide outstanding rate capability and long-term stability, retaining reversible capacities of 300–500 mAh g^−1^ over thousands of cycles.^85^ This is due to their expanded interlayer spacing and highly conductive surfaces, facilitating fast ion intercalation—crucial for high-power applications. Their usefulness extends to multivalent systems, effectively preventing zinc and aluminum dendrite formation and boosting ion transport at electrode interfaces. The materials' adjustable interlayer chemistry and high charge density make them especially suited for hosting divalent and trivalent ions.

Beyond individual applications, MXenes excel in hybrid systems. When combined with polymers or metal oxides, they form composites that integrate the strengths of batteries and supercapacitors—offering greater stability, higher capacitance, and enhanced mechanical properties. These hybrid configurations push energy storage technology forward, enabling devices with unmatched energy and power densities.

• Structural properties: MXenes typically possess surface groups such as F, OH, and O after being extracted from the MAX phase. Among these terminations, O- and OH- terminated MXenes are considered the most stable, as F terminations tend to transform into OH groups through rinsing or water storage. Notably, OH terminations can be further transformed into O terminations through high heat or metal adsorption processes. However, O-terminated MXene may break down into bare MXene when in contact with certain metals like Mg, Ca, or Al. Understanding the stability of different surface terminations is crucial in tailoring MXene properties for specific applications.^83,85^

• Electric properties: the electric properties of MXenes depend on their composition and surface terminations. Bare MXene species with the general formula Ti_*n*+1_X_*n*_ exhibit metallic behavior. However, as the value of “*n*” increases, the metallic properties become weaker due to the addition of Ti–X bonds. For example, titanium nitrides generally have stronger metallic properties than titanium carbides due to the N atom having one more electron than the C atom. On the other hand, terminated MXene sheets can exhibit either narrow-band-gap semiconducting or metallic behavior, depending on the type and orientation of surface groups. The tunability of the electronic structure in MXenes is essential for applications in optoelectronics and optics, where achieving a direct band gap is of particular interest.^82^

• Chemical properties: MXenes can undergo oxidation in certain environments, leading to the formation of TiO_2_ nanocrystals embedded in amorphous carbon sheets (TiO_2_–C hybrid structure). The oxidation mechanism can result in the formation of different TiO_2_ phases, such as anatase or rutile, depending on factors like reaction time and temperature. These chemical properties provide valuable insights into the stability and reactivity of MXenes, influencing their potential applications in catalysis, energy storage, and other fields.^86,87^

#### 2.4.2. Different synthesis methods of MXenes

MXenes are synthesized from MAX phases through a series of chemical reactions that involve dissolving the MAX phase in specific acids. The process requires precise corrosion times and agitation to break the M–A bonds and remove the A atom layers, resulting in the formation of MXenes, which are transition metal carbides, nitrides, or carbonitrides. One of the first MXenes to be synthesized was Ti_3_C_2_T_*x*_, achieved by treating Ti_3_AlC_2_ powders with 50% HF for 2 hours at room temperature. This process replaced the Ti_3_AlC_2_ with Ti_3_C_2_T_*x*_, as confirmed by X-ray diffraction (XRD) patterns.^84^

The XRD pattern after HF etching displayed shifts that were consistent with the presence of functional groups in the resulting MXene. The method of HF etching was successfully applied to various other MAX phases, leading to the discovery of numerous new MXenes with different compositions, such as Ti_2_CT_*x*_, V_2_CT_*x*_, Nb_2_CT_*x*_, Ti_2_NT_*x*_, (V_0.5_Cr_0.5_)_3_C_2_T_*x*_, (Ti_0.5_Nb_0.5_)_2_CT_*x*_, Mo_4/3_CT_*x*_, Nb_4/3_CT_*x*_, W_4/3_CT_*x*_, Ti_3_C_2_T_*x*_, Ti_3_CNT_*x*_, Nb_4_C_3_T_*x*_, Ta_4_C_3_T_*x*_, V_4_C_3_T_*x*_, Mo_2_TiC_2_T_*x*_, and Cr_2_TiC_2_T_*x*_.^84,88,89^

The distinction between M–X and M–A bond strengths is crucial for the synthesis of MXenes. The weaker M–A bonds allow for the removal of A atom layers from the MAX phase, leaving behind the desired carbides, nitrides, or both. The initial synthesis process involved treating the MAX phase with HF and exfoliating the resulting multi-layered structure through ultrasonication in alcohol solvents. The chemical reactions during the synthesis led to MXene termination with hydroxyl, fluorine, and later oxygen atoms, replacing the original Ti–Al bonds.

The prepared MXene samples typically consist of stacked multilayer flakes held together by weak interlayer interactions, allowing for easy exfoliation through ultrasonication. The discovery and synthesis of various MXenes have opened up exciting possibilities for their applications in diverse fields, including energy storage, catalysis, electronics, and more. Continued research and exploration in the field of MXenes hold the potential for further advancements and novel applications of these fascinating 2D materials.^88,90,91^

MXene is a new family of high performance electroactive material that have been shown to be good electrodes because of there excellent electrical conductivity and highly hydrophilic surface properties. Ti_3_C_2_T_*x*_ MXene thin film was able to demonstrate one of the highest reported values of volumetric capacitance (*i.e.*, 1500 F cm^−3^) at low scan rates in aqueous supercapacitor systems.^92^ The enhanced lithium storage ability of the MXene/graphene hybrid was also demonstrated by achieving 320 mAh g^−1^ at 1 A g^−1^ through the improvement of structural stability and enhancement of ion diffusion.^93^ Furthermore, Ti_3_C_2_T_*x*_ based supercapacitors were able to exhibit long term cyclic durability with 70% capacitance retained over 100 000 charge/discharge cycles, indicating that these materials are suitable for high frequency applications.

### 2.5. Molybdenum disulfide (MoS_2_)

Transition Metal Dichalcogenides (TMDs) are an intriguing class of 2D materials with the formula MX_2_, where M is a transition metal like molybdenum or tungsten, and X is a chalcogen such as sulfur or selenium. They have a distinctive sandwich-like structure, with metal atoms between two chalcogen layers (S–M–S or Se–M–Se), held together by weak van der Waals forces. This allows for easy mechanical or chemical exfoliation into atomically thin sheets. TMDs are especially interesting because their electronic properties can be tuned—they can act as semiconductors (MoS_2_, WS_2_), metals (NbSe_2_), or superconductors, depending on their composition and layer thickness. Their versatile electronic features, large surface area, and ability to intercalate ions make them highly promising for energy-related applications. They are used as high-capacity battery electrodes with enhanced cycling stability, contribute to pseudocapacitive energy storage in supercapacitors, and serve as efficient electrocatalysts for hydrogen production. Molybdenum disulfide (MoS_2_) is a well-studied example, widely researched for uses ranging from lithium-ion batteries to hydrogen evolution, because of its well-understood properties, ease of synthesis, and strong electrochemical performance across various energy systems.^85–87^

Molybdenum disulfide (MoS_2_) is an inorganic compound that belongs to Transition metal dichalcogenides (TMDCs). It consists of two atoms of sulphur and one atom of molybdenum. Transition metal dichalcogenides are chemical compounds consisting of a transition metal (Groups 4–12) and a chalcogen (Group 16). The monolayer MoS_2_ is a semiconductor with bandgap of 1.8 eV and its distinctive structure makes it a promising material to substitute graphene and making it feasible for 2D materials to be used in the future and optoelectronic devices. MoS_2_ is an excellent material for making thin-film transistors, because its fabrication is simple which means large production yield and low cost. The covalent bonds between molybdenum and sulphur and the van der Waals bonds between its layers make it optimal for gas sensing. MoS_2_ has achieved primary progress in the fields including energy conversion and storage and hydrogen evolution reaction (HER). In addition, MoS_2_ with odd number of layers could produce oscillating piezoelectric voltage and current outputs, indicating its potential applications in powering nano-devices and stretchable electronics.^86,94^

#### 2.5.1. Properties and structure of MoS_2_

The chemical formula of MoS_2_ is MX_2_ and where M means transition metal and X means chalcogen, and their family belongs to super-conducting, semiconducting and metallic. The single layer of MoS_2_ is arranged in a sequence of S–Mo–S by valent bonds in a sandwich like structure (M (+4) S(−2)). Although the relatively weak van der Waal forces influences the interaction of the sandwich like structure and these sandwich layers are approximately 0.65 nm in thickness.^95^

**Fig. 5:**
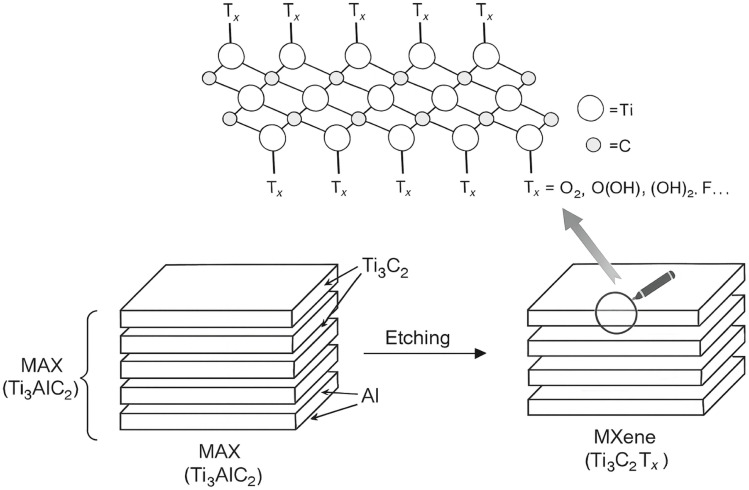
MXene preparation by MAX phase etching.

**Fig. 6:**
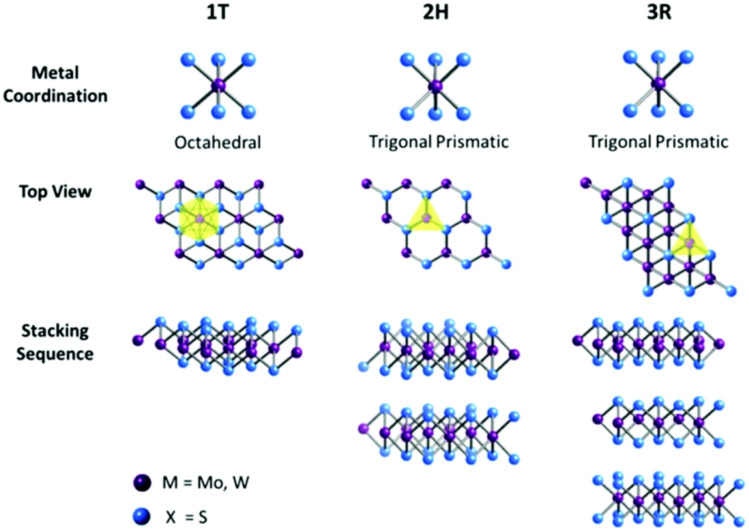
Different coordination and stacking sequences of the three MoS_2_ structures 1T, 2H and 3R reproduced from ref. 97 R. J. Toh, Z. Sofer, J. Luxa, D. Sedmidubský and M. Pumera, *Chem. Commun.*, 2017, 53, 3054, © 2017. The authors, licensed under CC BY 3.0.

The bulk (3D) structure of MoS_2_ possibly tetragonal (T), hexagonal (H) and rhombohedral (R), where 2H MoS_2_ means 2-layer hexagonal shape MoS_2_. MoS_2_ has basically three main structures such as 1T, 2H and 3R, where the 1T is octahedral structure and 2H and 3R is triagonal prismatic in structure (Fig. 5). The 1T structure is metallic and 2H, 3R are semiconducting and these two are used as dry unguent (Fig. 6).^96^

Every TMD polymorph possesses incomparable structures and electrical properties from where dissimilar catalytic properties are able to come out. Hence, it is crucial to make a contrast of distinct TMD polymorphs, intending to understand the effect of polymorph structure on the catalytic characters of TMD materials. When TMD materials subjected to chemical exfoliation, 2H → 1T phase change occurs and the catalytic characteristic of 1T and 2H has been exfoliated. The metallic 1T phase of MoS_2_ exhibits a greater level of HER activity than does the semiconducting 2H phase of MoS_2_, as a result of increased conductivity, more accessible surface sites and thinner flake size. This was explicated by the enhanced edge-exposed surface, reduced sheet size and improved electrical conductivity of the 1T phase.^98^

Transition Metal Dichalcogenides (TMDs), such as MoS_2_, feature a distinctive layered structure with van der Waals gaps that enable efficient ion intercalation and diffusion. This behaviour is similar to graphite but offers additional functionalities. Their electronic properties differ significantly between phases: the naturally occurring 2H phase acts as a semiconductor suitable for optoelectronics, while the chemically induced 1T phase becomes metallic, greatly increasing conductivity and electrocatalytic activity. With a very high specific surface area, these materials enhance electrolyte contact, improving charge storage. Their tunable bandgap and defect chemistry also allow precise manipulation of charge transport and redox processes.^87^

In lithium and sodium-ion batteries, MoS_2_'s layered structure enables efficient Li^+^/Na^+^ insertion, providing theoretical capacities close to 670 mAh g^−1^. When combined with conductive additives like graphene or carbon nanotubes, it achieves impressive cycling stability, with the metallic 1T phase offering better rate capability compared to its semiconducting form. For supercapacitor applications, MoS_2_ delivers substantial pseudocapacitance through reversible redox reactions at molybdenum centres, often exceeding 300 F g^−1^ when nanostructured into flower-like shapes or combined with conductive supports.^98^ These materials excel in hybrid devices, where MoS_2_-based composites combine faradaic storage with electric double-layer capacitance, creating asymmetric systems that optimise energy and power density while maintaining mechanical strength. The ability to precisely control phase, morphology, and composite formation makes MoS_2_ a highly versatile platform for next-generation energy storage technologies.

• Optical properties: the response of a material when a certain wavelength passes through it can be determined by the absorption coefficient and refractive index. The distance of spectrum passes inside the material before getting absorbed is determined by absorption coefficient. If the absorption coefficient is high, the attenuation to the wave applied will be high. The semiconductors have high absorption coefficient for short wavelengths and low absorption coefficient for long wavelengths. MoS_2_ has a comparatively large absorption coefficient for the wavelengths from 400 nm to 500 nm with a sharp decay at 500 nm. The reason behind the wide use of MoS_2_ in optoelectronics is its tunable bandgap that changes with size and structure; different bandgaps mean tunable photoresponsivity (R), specific detectivity, and response time, and thus, a wide range of applications. The MoS_2_ multilayers and monolayers have a high refractive index of more than 2, where it can be used in coating. Since the photoluminescence (PL) spectra are affected by the band gap, doping, and structure of the material, MoS_2_ has different PL activity. It has a peak exciton (A) in a single layer MoS_2_. The PL properties of monolayer MoS_2_ are enhanced by adding H_2_O_2_ solution, where it acts as a strong oxidizer without changing the crystalline structure of MoS_2_.^83,86,98^

• Electronic properties: the multilayer MoS_2_ has an indirect band gap of 1.2 eV, which increases with decrease in the number of layers until we get a monolayer of MoS_2_ with a direct band gap of 1.8 eV. However, band gap value of MoS_2_ is excellent. Due to the mechanical strain the band gap of MoS_2_ changes from direct to indirect band gap and transfers the material from a semiconducting material to metallic.^99,100^

The properties of MoS_2_ are determined by the 4d and 3p orbitals in Mo and S respectively. For the bulk and monolayer MoS_2_, the projected density of state is nearly the same but in the monolayer it shows some peaks. When the monolayer MoS_2_ is doped with chromium, copper and scandium it changes to an n-type semiconductor and to a p-type when doped with nickel and zinc. Doping with titanium (Ti) transfers MoS_2_ to a p-type or n-type semiconductor according to the levels and sites of doping. At low doping levels of Ti below 2.04%, MoS_2_ behaves as a p-type. In case of interstitial doping of Ti at 3.57% doping levels, the covalent bond between MoS_2_ and Ti are strong which increases the surface dipole moment that induces a reduction in electron affinity of 0.49 eV, where it behaves as an n-type. At high doping levels of 7.69%, the Fermi level shifts towards the conduction band and merges into the conduction band, where the surface dipole moment declines and the electron affinity rises, pinning Fermi level over the conduction band. In this case, MoS_2_ changes to a ferromagnetic half-metal with spin polarization equals to 1, that is promising for spintronics. On the other side the substitutional doping of Ti did not show any change in electronic properties for the three doping concentrations (2.04%, 3.57%, 7.69%).^89^

#### 2.5.2. Different synthesis methods of MoS_2_

Hydrothermal method under acid condition is used to synthesize MoS_2_ materials with distinct structure. Here molybdenum oxide (MoO_3_) and potassium thiocyanate (KSCN) were used as the starting materials. In this synthesis process, 1.5 mmol of MoO_3_ (0.2159 g) and 4.5 mmol of KSCN (0.4373 g) were dissolved in 40 mL of deionized water and sonicated for 30 min. After the first step, by adding 1 mol L^−1^ HCl the pH value of the solution is reconciled to 2.0, after that this solution is stirred for 30 min. For hydrothermal treatment, the well mixed solution was transferred to a 50 mL Teflon-lined stainless-steel autoclave about 240 °C for 37 h and cooled to room temperature. After cooling, the solution was centrifuged at 4000 rpm for 10 min, a black powder of MoS_2_ was collected. The black powder is then washed with deionized water and ethanol several times and dried in the vacuum oven at 60 °C for 12 h.^101^

**Fig. 7:**
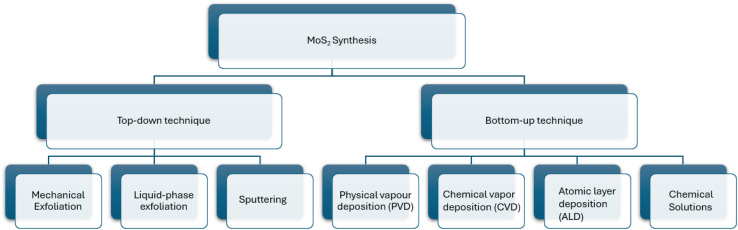
Different synthesis methods of MoS_2_.

Top-down methods and bottom-up methods are used to synthesize MoS_2_, and its end result is in different shapes, size and quantity. The top-down method is actually decided by engraving crystal planes from a substrate that has the crystals set down over it, and the crystals are layered over the substrate in the bottom-up method. The top-bottom methods to get MoS_2_ layers is by exfoliation. The weak van der Waals forces between layers of TMDs clear the way at the fore of disparate exfoliation synthesizing methods. A sticky tape is used to rub out the MoS_2_ by mechanical exfoliation and the MoS_2_ flakes were over it. This method is practically useful in laboratory synthesis but the yield is low. Liquid phase exfoliation is possible by adding chemicals and stirring, foaming or by milling. Liquid phase exfoliation is easy, quick economical and finish in 30 minutes, the carbon aerogels were used to avert the low yield of this method. This method enhances the electrical conductivity and porosity of MoS_2_ and avert the pyrophoric materials that are commonly used in liquid exfoliation. The dangerous materials used in the liquid phase method were cast out by sonication and this technique is easiest. In the sonication from a probe in a shape of bubbles ultrasonic waves produced and when it burst MoS_2_ layers will peel. The MoS_2_ nanosheet produced by sonication have dispute in practical applications. Commonly, top-down methods are said to have low controllability, and scalability and high cost. When using ultrasound sonication with supercritical carbon in with an intercalating solvent *N*-methyl-2-pyrrolidone (NMP) enhances yield >90% (Fig. 7).^83,86,98^

• Physical layer deposition (PVD) is a bottom-up method which is applied only in thin layer of MoS_2_ and grain size are variable. This method includes ion implantation like molecular beam epitaxy (MBE).

• Chemical vapour deposition (CVD) method is possible to thin and thick layers, where Mo is laid over a substrate and sulfur vapor passes over it. This method has good quality, but low yield.

• The atomic layer deposition (ALD) method is used to fabricate thick and thin films. The method is considered efficient and the layers have fewer impurities that can be used in different applications, including electronics and sensors.^102^

#### 2.4.3. MoS_2_ dispersion preparation

MoS_2_ dispersions were created by liquid phase exfoliation as has been reported previously.^86,98^ Briefly, MoS_2_ powder (10 mg mL^−1^) was dispersed in *N*-methyl-2-pyrrolidone (NMP) and ultrasonically processed in a bath sonicator (Elmasonic P70H) operating at 37 kHz and 40% amplitude for 12 h while cooling to maintain a stable temperature of 20 °C. After this sonication, the dispersions were centrifuged at 6000 rpm (3139 g) for 30 min to remove any unexfoliated material, the supernatant was then decanted and fresh solvent added before repeating the centrifugation to ensure a narrow distribution of flake dimensions and thickness. The resultant MoS_2_ suspensions were stable in solution for several months with no detectable sedimentation.

Because of their layered nature, and the ability to tune phases, MoS_2_ and other TMDs are demonstrating promising energy storage properties. MoS_2_ nanosheet anodes (in 1T phase) reached a capacity of 700 mAh g^−1^ after 200 cycles for LIBs, due to their electrically conductive nature and availability of many sites on which electrochemical reactions may occur.^103^ In addition, supercapacitor electrodes made with MoS_2_/rGO composites had a capacitance of 280F g^−1^ at a discharge rate of 1 A g^−1^ at low frequency (due to improved charge transport and less restacking).^104^ Also, electrodes based on WS_2_ nanotubes were able to demonstrate long-term stability, maintaining 90% of their capacity over 5000 cycles and thus proving that materials with TMD structures can be durable.^105^

## 3. Characterisation techniques for 2D materials

Material characterization plays a crucial role in advancing energy storage technologies, as the performance of batteries and supercapacitors largely depends on their material properties. Examining structural, chemical, and electronic characteristics helps optimise efficiency, stability, and energy density. Without accurate characterization, it becomes difficult to connect material traits with device performance, potentially hindering progress in developing new energy storage solutions.

Analyzing 2D materials such as graphene and TMDs presents particular challenges due to their atomic-scale thickness, surface defects, and structural variability. Their ultra-thin nature makes them susceptible to environmental influences, complicating measurements. While defects can sometimes boost catalytic activity, they may also unpredictably affect electrical and mechanical properties. Variations in layer stacking and chemical composition require high-resolution techniques for detailed analysis, as standard methods might overlook critical nanoscale features.

**Fig. 8:**
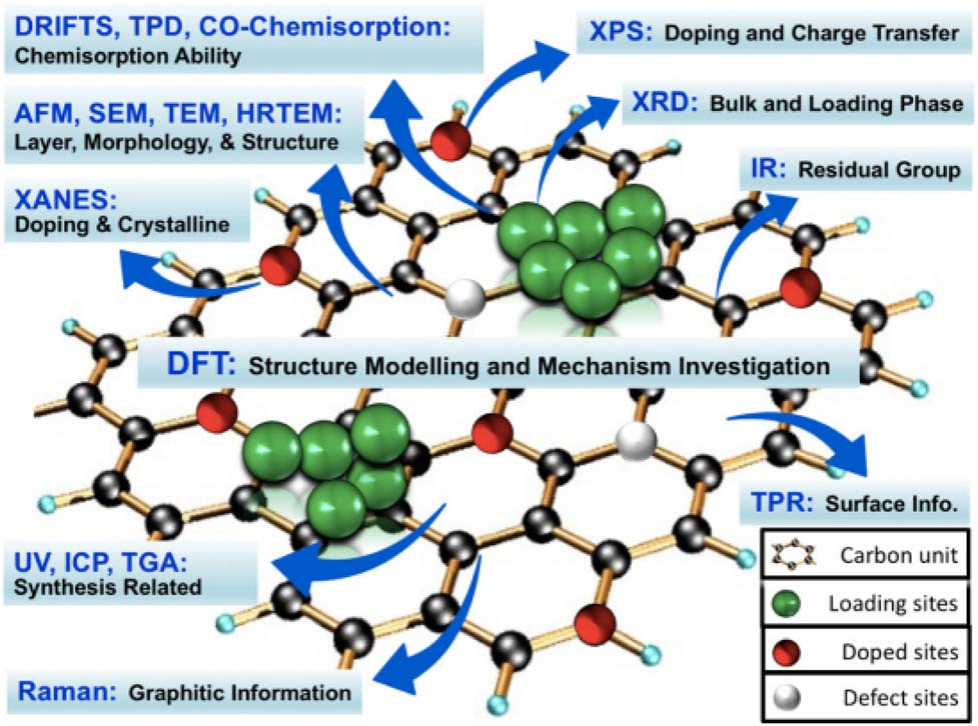
Different characterisation techniques for graphene based materials reproduced from ref. 106 with permission from John Wiley and Sons, *ChemistrySelect*, S. B. Singh and C. M. Hussain, 2018, **3**, 12, © 2018 John Wiley and Sons.

Different characterization techniques provide insights tailored to specific energy applications. For batteries, methods like X-ray diffraction (XRD) and electron microscopy are used to observe phase changes and degradation across cycles. In supercapacitors, surface area measurements and electrochemical impedance spectroscopy (EIS) assess charge storage mechanisms. For hydrogen evolution reaction (HER) catalysts, spectroscopy and microscopy help identify active sites and surface characteristics. Selecting the right method is key to addressing material-specific challenges and improving device performance (Fig. 8).

### 3.1. Spectroscopic techniques

#### 3.1.1. Raman spectroscopy

##### 3.1.1.1. Graphene

**Fig. 9:**
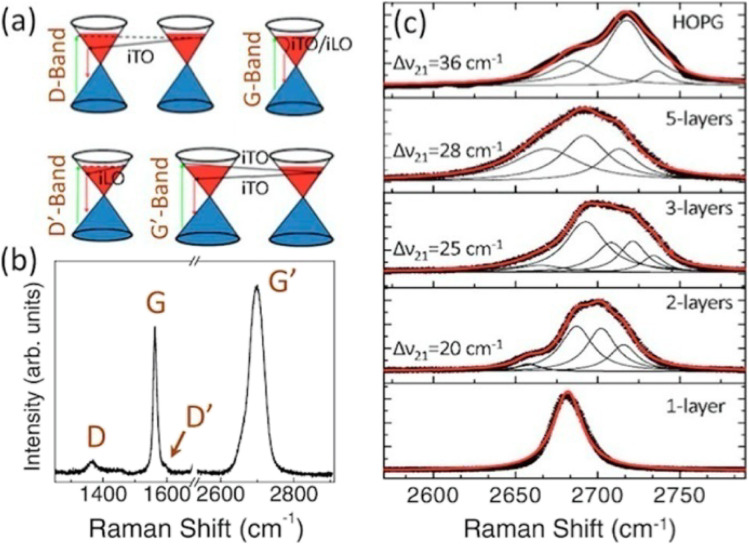
Raman spectra of graphene: (a) schematic illustration of the D, G, D′ and G′ (2D) band scattering processes; (b) representative Raman spectrum showing the D, G, D′ and G′ bands; (c) evolution of the G′ band with increasing number of layers (1-layer to HOPG). Reproduced from ref. 111 with permission from AIP Publishing, *AIP Conference Proceedings*, N. M. S. Hidayah and W.-W. Liu, 2017, 1892, **1**, © 2017 AIP Publishing.

Graphene has six normal modes and two atoms per unit cell at the Brillouin zone center (*Γ*). The E_2g_ phonons are particularly active in Raman spectroscopy. The three most distinctive features of graphene's Raman spectrum are the D peak (1320–1350 cm^−1^), G peak (1580–1605 cm^−1^), and 2D (or G′) peak (2640–2680 cm^−1^).^107,108^ The D mode is associated with defects and disorder within the graphite layers, while the G and 2D bands represent the hexagonal lattice structure. The D′ peak (around 1602–1625 cm^−1^) may also be present and is linked to defects and a double-resonance process. These characteristic peaks can be studied in graphene-based catalysts, where some parameters may change due to factors such as interaction with the catalyst, number of graphene layers, heteroatom doping, and strain (Fig. 9).^109,110^

##### 3.1.1.2. Reduced graphene oxide (rGO)

Raman spectroscopy (RS) is an invaluable technique for the analysis of Graphene (G) as it provides crucial information about the number of G layers and the presence of defects within the sheets. Moreover, it holds promise as a tool for characterizing the reduction of Graphene Oxide (GO) to Reduced Graphene Oxide (rGO).

In past studies, distinct characteristic peaks have been observed in G's Raman spectrum at around 1600 and 2700 cm^−1^, corresponding to the graphitic (G) and 2D bands. The G band arises from the vibration of sp^2^ hybridized carbon atoms in the honeycomb lattice structure of G, while the 2D band originates from the Raman scattering of second-order zone boundary phonons in G. The intensity, position, and shape of these bands are pivotal in estimating the number of G layers. Notably, the shape (intensity and width) and position of the 2D band show a trend of increasing with an increasing number of stacked G layers.^107,112,113^

**Fig. 10:**
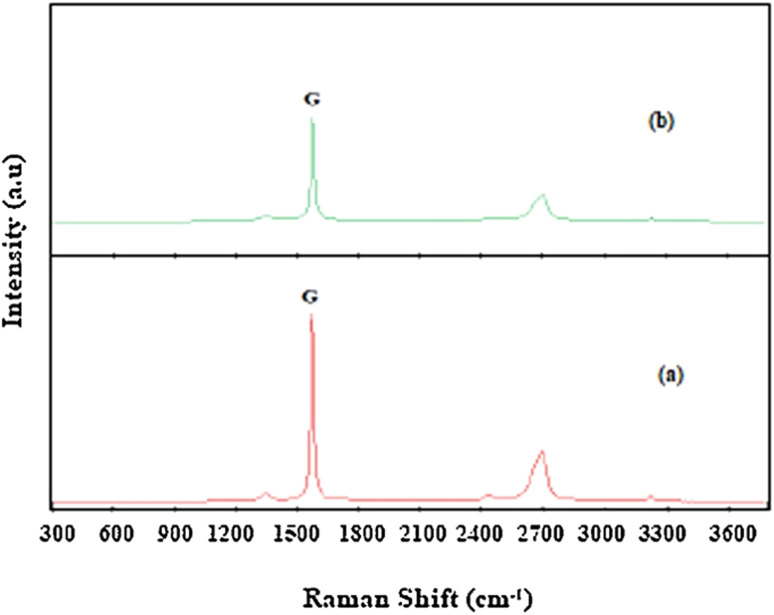
Raman spectra of graphene oxide (GO) and reduced graphene oxide (rGO): (a) GO; (b) rGO, showing the characteristic D (∼1350 cm^−1^) and G (∼1580 cm^−1^) bands. Reproduced from ref. 114 with permission from Elsevier, *Journal of Materials Research and Technology*, R. Ikram, B. M. Jan and W. Ahmad, 2020, **9**, 11587–11610, © 2020 Elsevier.

Additionally, a weak defect band (D) is evident around 1400 cm^−1^ in the Raman spectrum of single-layer G, indicating the presence of disorder, defects, or edge sites. This defect band provides valuable insights into the structural properties of graphene-based materials, revealing information about the quality and integrity of the graphene sheets^109^ (Fig. 10).

##### 3.1.1.3. Molybdenum sulfide (MoS_2_)

**Fig. 11:**
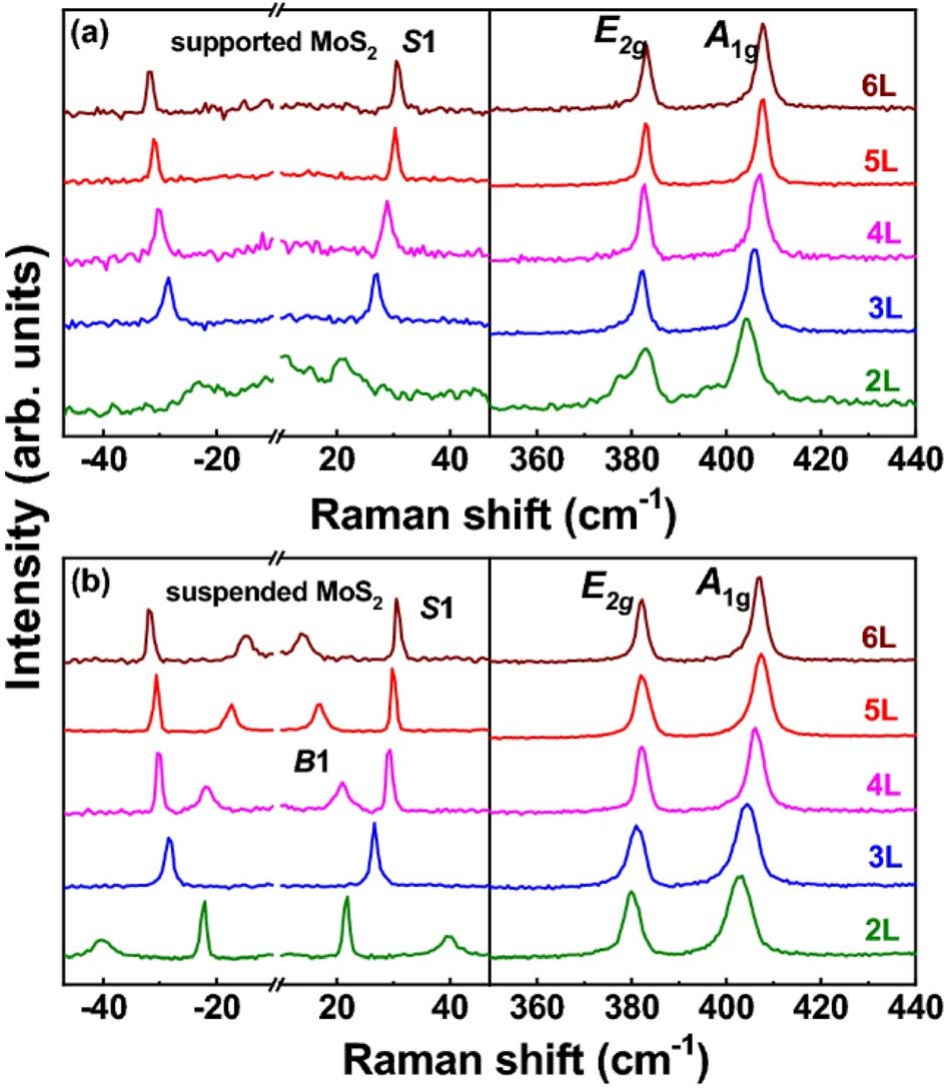
Temperature-dependent Raman spectral analysis of few-layer MoS_2_: (a) supported MoS_2_ and (b) suspended MoS_2_ with layer numbers from 2L to 6L, showing the evolution of the low-frequency shear (S1/B1) modes and the high-frequency E^1^_2g_ and A_1g_ phonon modes. Reproduced from ref. 115, Z. Lin, W. Liu, S. Tian, K. Zhu, Y. Huang and Y. Yang, *Scientific Reports*, 2021, **11**, 7037, © 2021. The authors (CC BY 4.0).

The crystal structure of the MoS_2_ samples was investigated using Raman spectroscopy Fig. 11. The Raman spectra presented in Fig. 11 display the characteristic vibrational modes of MoS_2_. Two prominent Raman peaks are observed at approximately 382 cm^−1^ and 406 cm^−1^, which correspond to the E^1^_2g_ (in-plane) and A_1g_ (out-of-plane) vibrational modes of Mo–S bonds in MoS_2_, respectively. The appearance of these two characteristic modes confirms the formation of crystalline MoS_2_. An increase in the Raman intensity of these Mo–S vibrational modes is observed, indicating enhanced crystallinity of the MoS_2_ samples. Additionally, a weak and broad band centered around 450 cm^−1^ is detected, which is commonly assigned to a second-order Raman scattering process associated with the longitudinal acoustic phonons at the M point of the Brillouin zone (LA(M) mode ≈ 227 cm^−1^). This second-order Raman mode is typically observed in bulk MoS_2_ and is attributed to strong electron–phonon coupling. In contrast, samples lacking distinct E^1^_2g_ and A_1g_ modes indicate the absence of well-developed crystalline MoS_2_.^115^

##### 3.1.1.4. MXene

MXenes constitute a broad class of two-dimensional transition-metal carbides and nitrides with a general formula M_*n*+1_X_*n*_T_*x*_, where M denotes an early transition metal, X represents carbon and/or nitrogen, and T_*x*_ corresponds to surface terminations such as –O, –OH, and –F. The Raman response of MXenes is highly sensitive to their structural features, including layer thickness, surface chemistry, oxidation state, and defect concentration.

The most prominent Raman features of MXenes are typically observed in the 100–800 cm^−1^ region, where multiple bands arise from lattice vibrations and interactions involving surface terminations. In particular, low-wavenumber peaks appearing in the range of approximately 100–240 cm^−1^ are attributed to collective lattice vibrations corresponding to E_g_ and A_1g_ modes. Variations in peak position and relative intensity within this region may indicate structural disorder, oxidation, or partial transformation of MXenes, including the possible formation of titanium dioxide (TiO_2_), as evidenced by the characteristic anatase-related feature near 150 cm^−1^.

**Fig. 12:**
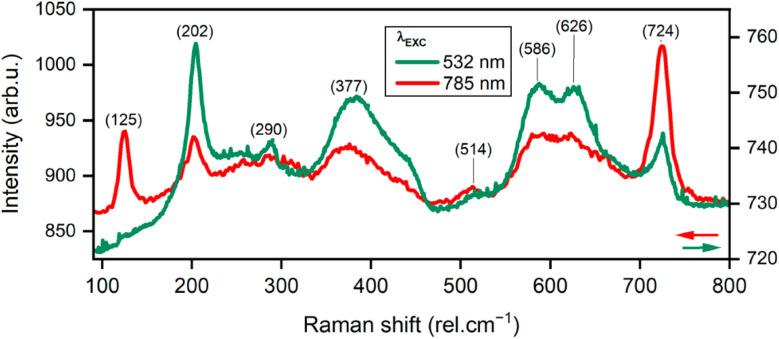
Correlation between surface morphology of Ti_3_C_2_T_*x*_ MXene-based substrates and their SERS performance, reproduced from ref. 116, F. Salehtash *et al.*, *Materials*, 2024, **17**, 1385, © 2024. The authors (CC BY 4.0).

Raman bands located in the 550–650 cm^−1^ range are associated with in-plane C–C vibrational modes of the carbon layers and are particularly sensitive to the nature and distribution of surface terminations such as –O, –OH, and –F. Oxidation processes typically result in a red shift of these bands, whereas an increased proportion of –O-terminated species can lead to a blue shift. At higher wavenumbers (1000–1800 cm^−1^), C–C vibrational modes provide insight into the carbon framework of the MXene films and often show significant variation due to differences in surface heterogeneity and structural disorder (Fig. 12).^116^

MXene Raman spectra can be difficult to interpret due to variations in peak position, shape and intensity based on the thickness of the MXene sample, the type and amount of surface terminating functional groups (–F, –O, –OH), defects and environmental conditions present. Changes in the chemical properties of the surface of MXene samples, such as oxidation, can cause significant shifts and changes in intensity of vibrational modes related to the lattice of MXene.

#### 3.1.2. Fourier transform infrared spectroscopy (FTIR)

##### 3.1.2.1. Graphene oxide and reduced graphene oxide

**Fig. 13:**
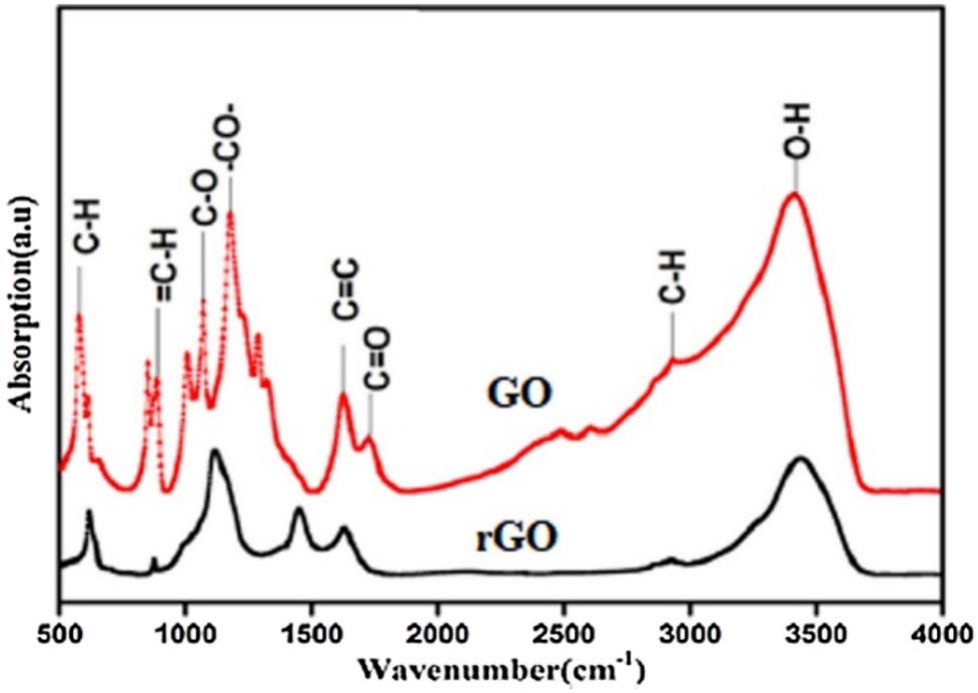
FTIR spectra of GO and rGO reproduced from ref. 117 with permission from John Wiley and Sons, *Macromolecular Symposia*, N. Sharma, V. Sharma and Y. Jain *et al.*, 2017, **376**, 1–5, © 2017 John Wiley and Sons.

Monteserin^118^ conducted an FTIR spectral analysis of GO, and rGO, as shown in Fig. 13. However, after the oxidation process, characteristic bands appeared in GO attributed to the absorption of oxygenated functional groups (Fig. 13).

In GO, a broad band at 3400 cm^−1^ indicated the O–H bond/stretching vibrations of water molecules and other hydroxyl functional groups. Bands at 2927 and 2849 cm^−1^ corresponded to the symmetric and asymmetric stretching of C–H in GO. Similar bands were observed in Gr, resulting from defects in the SP2 matrix of the graphitic plane, which could also be seen in the Raman spectrum of G. However, the intensity of these bands in GO was significantly higher than in Gr, indicating a higher number of C–H functionalities.^119,120^

The bands at 1743 and 1210 cm^−1^ were attributed to the C=O and C–O stretching vibrations of –COOH groups at the edges of GO sheets, respectively. In rGO, the intensity of all the above-mentioned bands reduced significantly due to the removal of oxygenated functionalities. Additionally, a few bands appeared at 1050 cm^−1^, resulting from the stretching vibrations of C–O–C from the epoxy groups.

In the FTIR spectra, rGO exhibited a narrower peak at 3420 cm^−1^ compared to GO, indicating a significant reduction in hydroxyl groups through the reduction process with hydrazine hydrate. Additionally, other peaks at 1739, 1226, and 1015 cm^−1^ in the FTIR spectra of rGO became less intense when compared to GO, providing further evidence of partial removal of oxygen functional groups.^121,122^

Despite the reduction process, low levels of residual functional groups were observed to remain at the edges and basal plane of rGO. These residual functional groups contribute to the stability and dispersion characteristics of rGO and can also serve as sites for potential chemical modifications or functionalization. The narrower peak at 3420 cm^−1^ and the reduced intensity of other peaks in the FTIR spectra of rGO indicate the successful removal of oxygen functional groups during the reduction process. This transformation from GO to rGO results in the restoration of sp^2^ hybridized carbon structures, leading to improved electrical conductivity and other desirable properties^121,122^ (Table 2).

**Table 2:** FTIR peaks for major functional groups with their wavenumbers

Wavenumber (cm^−1^)	Functional group
3400	O–H stretching (water and hydroxyl)
2927, 2849	C–H stretching (symmetric and asymmetric)
1743	C=O stretching (carboxylic acid)
1210	C–O stretching (carboxylic acid)
1050	C–O–C stretching (epoxy groups)

##### 3.1.2.2. MXene

**Fig. 14:**
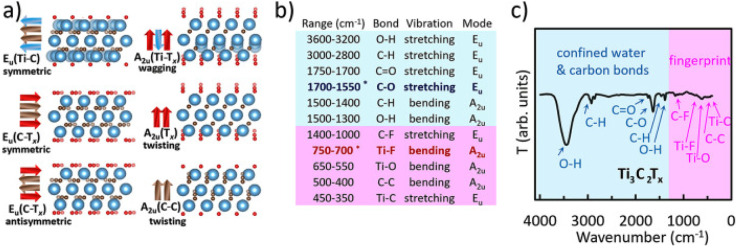
FTIR spectroscopy characterization of Ti_3_C_2_T_*x*_ MXene. (a) E_u_ and A_2u_ vibration modes (the images of molecular structures were generated in VESTA),^124^ (b) FTIR peak positions, and (c) FTIR spectrum with assigned bond vibrations.^123^ Reproduced from ref. 123 T. Parker, D. Zhang, D. Bugallo, K. Shevchuk, M. Downes, G. Valurouthu, A. Inman, B. Chacon, T. Zhang, C. E. Shuck, Y. J. Hu and Y. Gogotsi, *Chemistry of Materials*, 2024, **36**, 8437–8446, © 2024. The authors (CC BY).

The FTIR data are consistent with the expected terminal functional groups present on the surface of MXenes (*i.e.*, typically –OH, =O, –F). The main bands include O–H stretching between 3600–3200 cm^−1^ from both –OH terminal groups and bound water; M–O bending (M = Ti, V) between 650–550 cm^−1^; M–F bending (M = Ti, V) between 750-700 cm^−1^; and C–F stretching between 1400–1000 cm^−1^. The bands that appear within the “fingerprint” region (1400–400 cm^−1^) are very sensitive probes of the termination chemistry and composition, with many exhibiting peak shifts as a result of the presence of mixed termination chemistries and coordinative effects. Bands corresponding to the O–H bending from the –OH terminal groups will appear between 1500–1300 cm^−1^ and thus can be used in conjunction with XPS to analyze the surface chemistry^123^ (Fig. 14).

#### 3.1.3. X-ray diffraction (XRD)

##### 3.1.3.1. Graphene

XRD commonly shows two peaks at 2*θ* of ∼26° for pristine graphite and ∼11° for graphene oxide in graphene-based catalysts. Nguyen^125^ used XRD to study the crystalline phase of frost-like CuO combined with graphene–TiO_2_ as a catalyst for photocatalytic degradation of organic pollutants. The results showed typical diffraction signals for graphene, CuO, and TiO_2_. The peak confirmed the graphene presence at 2*θ* of 25.9° for (002) reflection in CuO–graphene and CuO–graphene–TiO_2_ composites. The peaks attributed to polycrystalline monoclinic CuO phase (110), (111), (202), (020), (311), (220), and (222) confirmed the loading of catalytic species. The existence of anatase TiO_2_ was supported by peaks for (101) and (200).

**Fig. 15:**
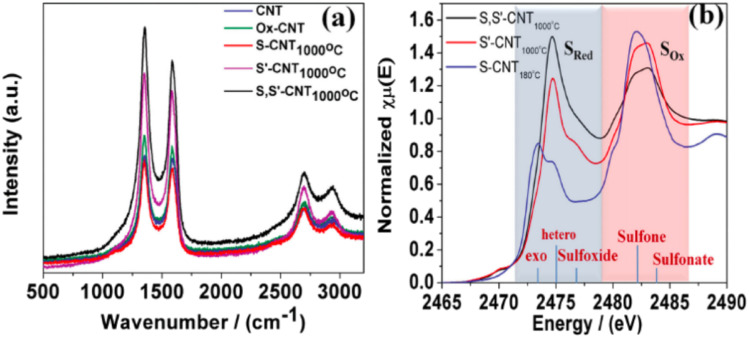
(a) Raman spectra and (b) sulfur K-edge X-ray absorption reproduced from ref. 111 with permission from John Wiley and Sons, *Advanced Energy Materials*, A. M. El-Sawy, I. M. Mosa, D. Su, *et al.*, 2015, **6**, 5, © 2015 John Wiley and Sons.

The purpose of using XRD to analyze graphene-based catalysts is to verify the transformation of GO to graphene.^126,127^ Reduced graphene oxide/g-C_3_N_4_ composite membranes were developed for catalytic degradation of organic contaminants and oil-in-water emulsion separation. XRD was applied to examine GO, rGO/poly-dopamine (PDA), g-C_3_N_4_, and rGO/PDA /g-C_3_N_4_ composites. The analysis of Raman spectroscopy (Fig. 15(a)) and sulfur K-edge XANES spectra (Fig. 15(b)) for the influence of sulfur functionalization and heat treatment on the structural and chemical characteristics of carbon nanotubes (CNTs) are shown in Fig. 15. The variation in the intensity of the D and G bands in Raman spectra for the three types of samples; CNT, oxidized CNT and sulfur treated CNT show varying defect densities and graphitization levels, while high temperature sulfur treatment (S-CNT_1000 °C_, S′-CNT_1000 °C_) leads to increased defects and changes to the carbon structure. The XANES Spectra support the incorporation of sulfur into different oxidation states (red S, sulfoxide, sulfone, and sulfonate) identified by unique absorption features. The evidence of multiple sulfur environment post heat treatment demonstrates the successful sulfur doping of CNTs, and illustrates how heat treatment processes affect the chemical state distribution of sulfur incorporated in CNTs. These studies provide an understanding of both the structural transformation and the interactions between sulfur and carbon that determine the electrochemical performance of CNT-based electrodes modified with sulfur.^111^

##### 3.1.3.2. Graphene oxide

The (001) lattice plane of GO is evident in its XRD pattern at a 2*θ* value of 10°, showing distinct peaks. These peaks indicate the considerable space between the graphene sheets due to electronegative functional groups, which prevent close stacking through π–π interactions. There are also studies on Bruker's D8 advanced X-ray diffractometer with monochromated Cu Kα radiation to analyse Gr flakes and GO.^128,129^

Upon reduction of GO to rGO, the peak at 2*θ* value of 10° disappears, replaced by two new peaks at 26.8° and 42.7°, attributed to (002) and (100) planes, respectively. These new peaks indicate the restacking of graphene sheets during the reduction process, resulting from removing oxygenated electronegative groups. This process allows for π–π interactions to take place.^130^ Neutron diffraction is another effective method for characterising the structure of bulk-scale graphene and its derivatives obtained through thermal or chemical reduction. Researchers have found neutron diffraction particularly valuable in evaluating particle size and the number of graphene layers compared to other characterisation techniques.^131^ However, utilising neutron diffraction for industrial-scale characterisation of graphene-based materials poses challenges. Despite this, it offers accurate and reliable information about the size and thickness of lateral graphene sheets, enabling improvements in characterisation techniques.

##### 3.1.3.3. Reduced graphene oxide (rGO)

The reduction of oxygen-containing functional groups in GO leads to a significant improvement in its π-conjugated structure and the restoration of the graphene crystal phase, as evident in the XRD patterns of rGO. The XRD pattern of rGO exhibits a broad peak at 2*θ* = 24.10°, indicating a remarkable restoration of the graphene structure. Furthermore, the *d*-spacing of rGO is reduced from 0.97942 nm to 0.36895 nm, providing clear evidence of the efficient removal of oxygen-containing functional groups during the reduction process.

Although the arrangement of the rGO crystal phase (002) is random compared to the high crystallization structure of graphite, this is attributed to the formation of only a few layers of rGO after reduction. The presence of strong van der Waals forces between layers in rGO also contributes to the formation of thick piles of rGO, resulting in a less ordered stacking arrangement.^132,133^

**Fig. 16:**
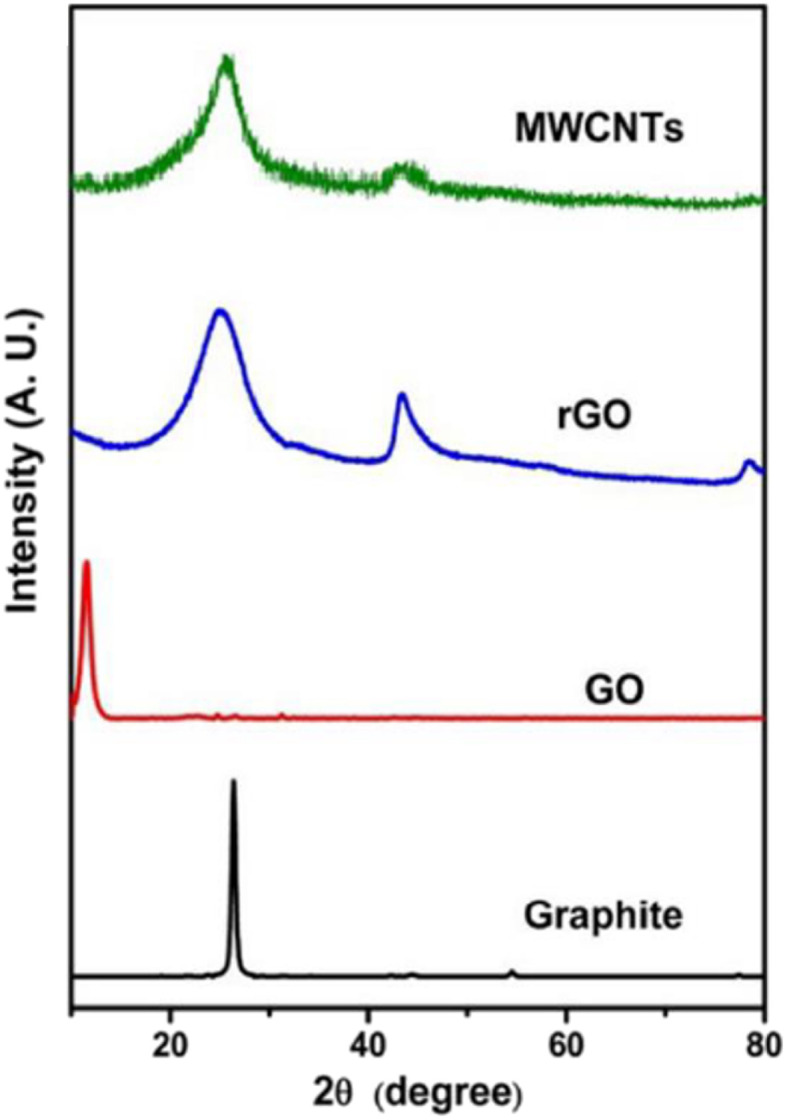
XRD spectra of graphite, GO and rGO reproduced from ref. 134 K. Sa, P. C. Mahakul, B. V. R. S. Subramanyam, J. Raiguru, S. Das, I. Alam and P. Mahanandia, *IOP Conference Series: Materials Science and Engineering*, 2018, **338**, 012055, © 2018. The authors (CC BY).

Another less intense peak is observed at 2*θ* = 42.60° with (001) orientation, which can be attributed to the turbostratic band of disordered carbon materials. This indicates the presence of some degree of disorder in the rGO structure. The restoration of the graphene crystal phase and the reduction in *d*-spacing in rGO are indicative of successful reduction and removal of oxygen-containing functional groups, resulting in a material with enhanced π-conjugation and improved electrical conductivity. The unique structural properties of rGO make it a versatile material with a wide range of potential applications, including electronics, sensors, energy storage devices, and composites^76,109^ (Fig. 16).

##### 3.1.3.4. MXenes

**Fig. 17:**
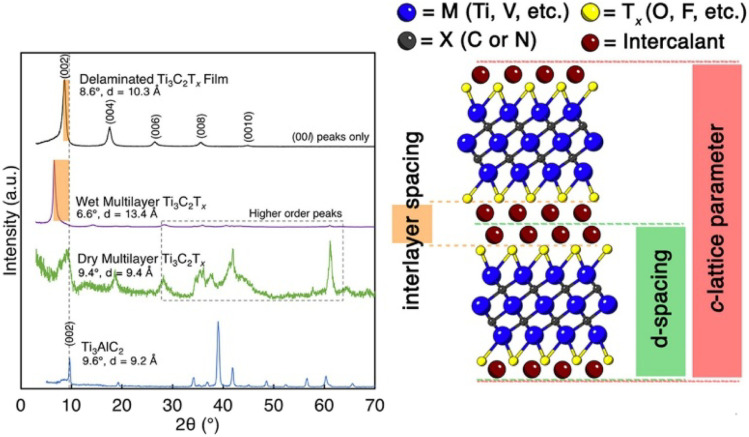
X-ray diffraction (XRD) patterns obtained for Ti_3_AlC_2_, dry multilayer Ti_3_C_2_T_*x*_, wet multilayer Ti_3_C_2_T_*x*_ immediately post-etching, and a vacuum-filtered film delaminated (with LiCl). On the right, a schematic illustrates variations in interlayer spacing (orange), *d*-spacing (green), and c-lattice parameter (pink), in comparison to a M_3_X_2_T_*x*_ structure featuring two layers of intercalant (*e.g.*, water, TMA^+^, Li^+^) reproduced from ref. 138 with permission from Elsevier, *Progress in Materials Science*, M. Shekhirev, C. E. Shuck, A. Sarycheva and Y. Gogotsi, 2021, **120**, 100757, © 2021 Elsevier.

XRD is a valuable and straightforward technique to confirm the production of MXenes. The XRD pattern undergoes changes during the transformation from MAX phases (*e.g.*, Ti_3_AlC_2_) to multilayer MXenes and then to delaminated MXenes. In the initial XRD pattern of MAX, all crystallographic peaks are observed, consistent with its *P*6_3_/*mmc* structure (Fig. 17.) As the MAX phase is processed into multilayer MXene, the XRD pattern of the dried powder shows broader and shifted (00l) peaks and fewer higher-order peaks compared to standard Ti_3_AlC_2_. In the hydrated form of multilayer powder, the higher-order peaks are reduced or absent compared to the (00l) peaks.^90,135^ During etching of Ti_3_AlC_2_, the amount of the Al related peaks that appear is decreased, and the (002) peak will move to a lower 2*θ* angle. The lower 2*θ* angle is indicative of an increased *d* spacing due to the termination of hydroxide/hydrogenfluoride (OH/O/F). When delaminated from the substrate it causes higher order peaks to be lost as the material has lost its long range order. These changes in XRD confirm that the MXene has been successfully created.

For delaminated MXene flakes in film form, only the (00l) peaks are present when the flakes are aligned in-plane. In 2D materials, it is common practice to report the (00l) spacing indices as (001), (002), (003), *etc.* because the films are made of randomly stacked identical 2D flakes with one sheet per unit cell. However, in the MXene community, a different indexing convention, such as (002), (004), (006), *etc.*, is more commonly used. This convention originates from their production from MAX phases, which have two layers (M_3_X_2_) per unit cell in a *P*6_3_/*mmc* structure. Although the stacking becomes random in delaminated flakes, the (002), (004), (006), *etc.* indexing is often retained to facilitate comparisons with MAX phases.

It's worth noting that referencing the c-lattice parameter or *d*-spacing as “interlayer spacing” can be somewhat misleading. The c-lattice parameter of MXene represents two MXene sheets and two interlayer spacings, whereas *d*-spacing only represents one MXene sheet and one interlayer spacing. To determine the interlayer spacing accurately, a two-step process is necessary: first, measure the *d*-spacing of the dry sample without intercalants, and then measure the XRD pattern in the active state (*e.g.*, wet multilayer powder or freestanding film). The change in *d*-spacing or half of the c-lattice parameter will indicate the presence of intercalants between the layers. Removing all intercalants can be challenging and may require annealing at high temperatures in a vacuum due to strong bonding.^136,137^

##### 3.1.3.5. Molybdenum sulfide (MoS_2_)

**Fig. 18:**
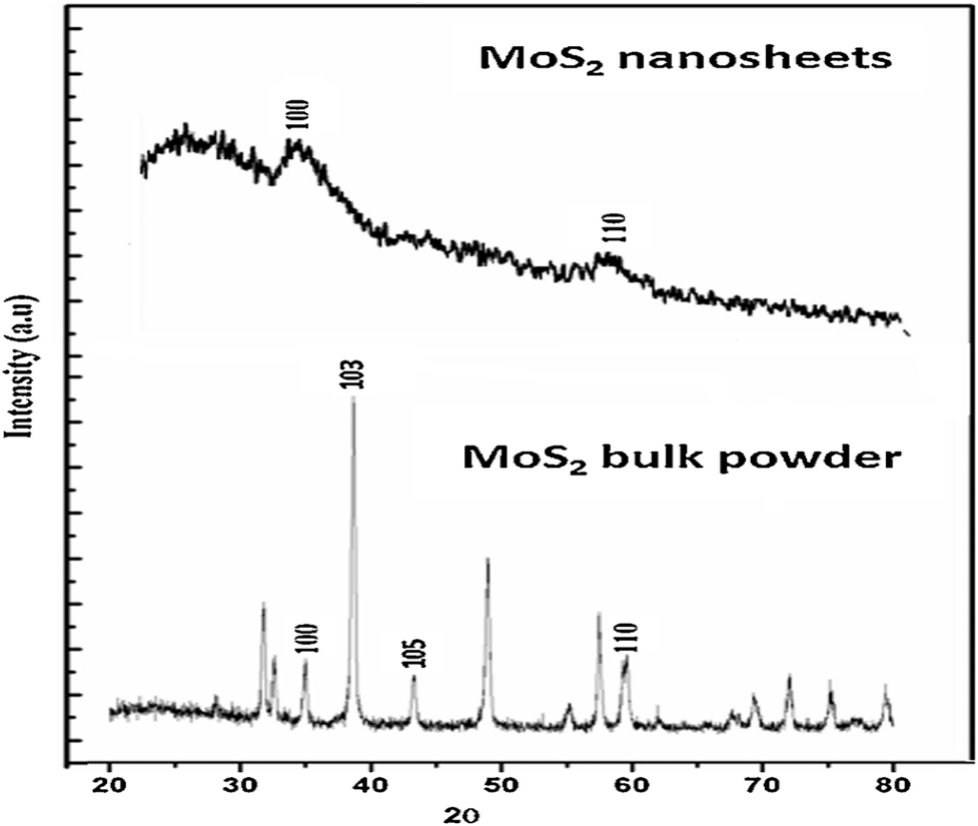
XRD peaks of MoS_2_ nanosheets reproduced from ref. 139 with permission from Elsevier, *Applied Surface Science*, C. P. Veeramalai, F. Li, Y. Liu, Z. Xu, T. Guo and T. W. Kim, 2016, **389**, 1017–1022, © 2016 Elsevier.

**Fig. 19:**
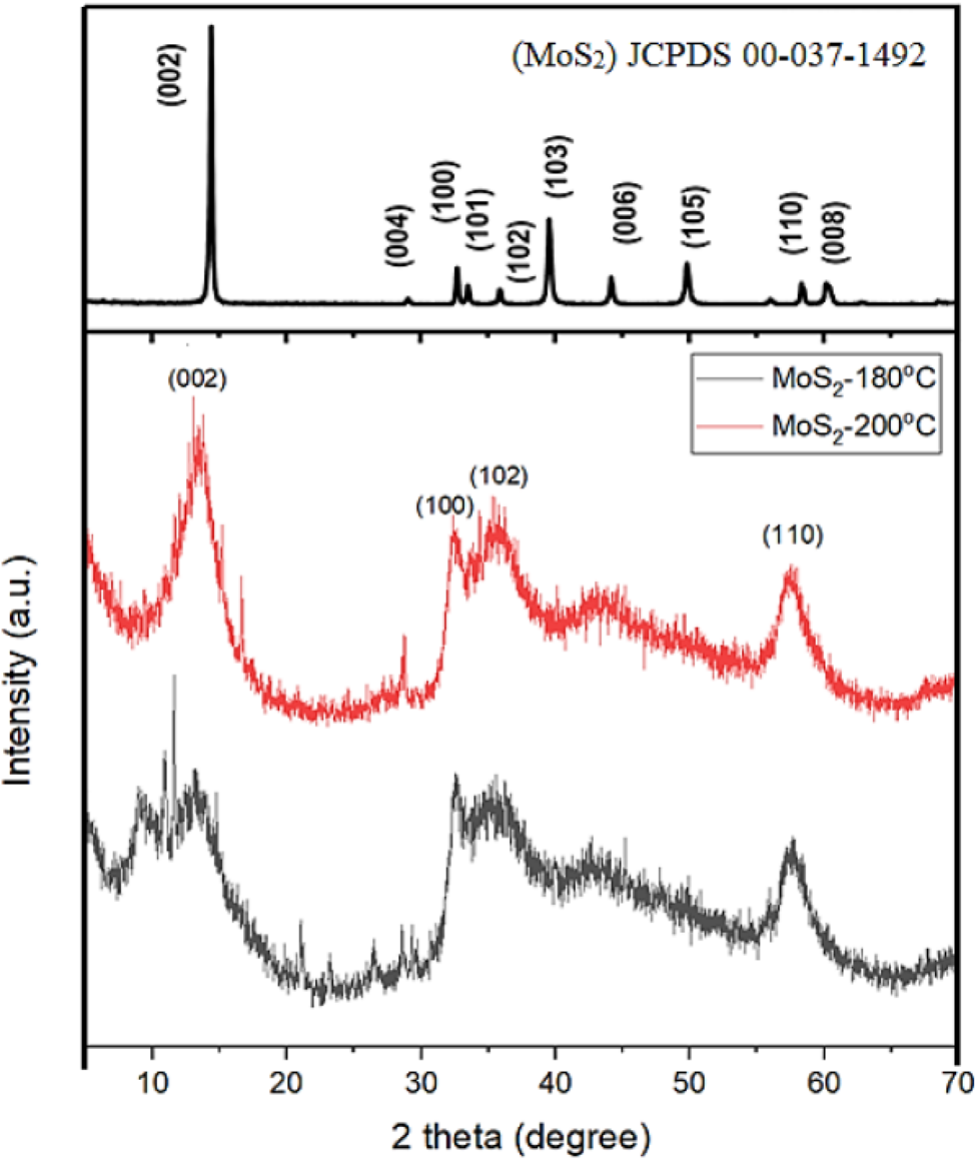
XRD patterns of MoS_2_ prepared at different temperatures reproduced from ref. 140 H. F. Hasmin, C. Imawan and V. Fauzia, *J. Phys.: Conf. Ser.*, 2021, **1951**, 012014, © 2021. The authors (CC BY).

In the (100) and (110) plane at 33.69° and 59.51° the characteristic peaks of MoS_2_ nanosheets were noticed. Because of the vacancy of (002) reflections and broadness of peaks in contrast with bulk MoS_2_ crystals specify that the outcome is single layer or few layer graphene like MoS_2_ (ref. 139) (Fig. 18 and 19).

The XRD patterns of MoS_2_ prepared under different hydrothermal temperatures (MoS_2_-180, and MoS_2_-200), are presented, showing that the crystallinity of MoS_2_ improves with increasing hydrothermal temperature. For the samples synthesized at approximately 150 °C and 180 °C, a weak diffraction peak is observed at a 2*θ* value of 32.91°, which corresponds to the (100) plane of MoS_2_, whereas the characteristic and strongest (002) diffraction peak at 14.39° is absent. This observation indicates poor crystallinity at lower temperatures. Consistently, FESEM images reveal the absence of a well-defined layered structure for these samples.

In contrast, for the samples obtained at 210 °C and 240 °C, diffraction peaks located at 2*θ* values of 14.39°, 32.91°, 39.69°, and 58.76° are clearly observed, which can be indexed to the (002), (100), (103), and (110) planes of hexagonal MoS_2_, respectively. These peaks are in good agreement with the standard diffraction data for 2H–MoS_2_ (*a* = *b* = 0.316 nm, *c* = 1.230 nm; JCPDS No. 37-1492). The (002) peak exhibits relatively higher intensity, which is attributed to the stacking of MoS_2_ layers in the MoS_2_-210-37 and MoS_2_-240-37 samples. No conspicuous diffraction peaks corresponding to impurity phases are detected. These results indicate that hydrothermal synthesis at temperatures above 210 °C favors the formation of phase-pure hexagonal MoS_2_ with improved crystallinity.^140^

#### 3.1.4. Ultraviolet spectroscopy

**Fig. 20:**
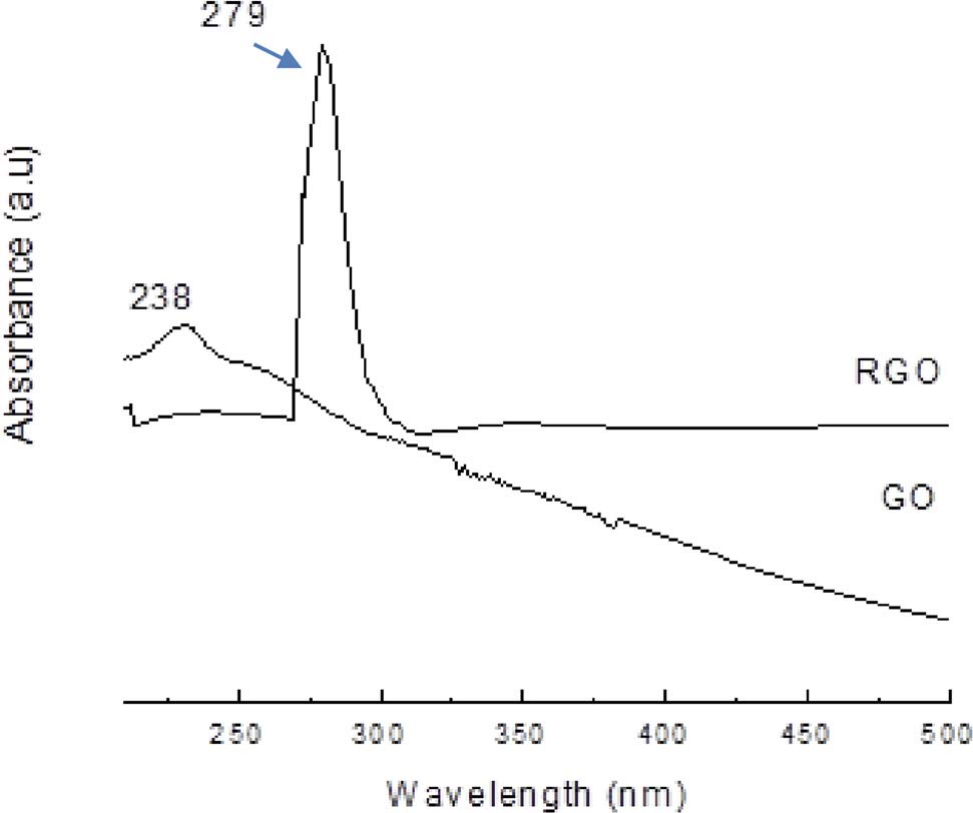
UV-Vis spectra of GO and rGO reproduced from ref. 144 with permission from AIP Publishing, *AIP Conference Proceedings*, N. M. S. Hidayah and W.-W. Liu, 2017, **1892**, 1, © 2017 AIP Publishing.

As shown in Fig. 20, the absorption peak of the Graphene Oxide (GO) dispersion was observed at 238 nm. This result closely aligns with the previously reported findings, where a similar absorption peak was detected at 231 nm. Li^141^ further observed a redshift of the absorption peak towards higher wavelengths (>231 nm) with increasing reaction times. This strong absorption peak around 230 nm is attributed to the π–π* transitions of graphitic C–C bonds.^122,142^

Following the reduction process, the rGO exhibited a redshift in its absorption peak from 238 to 279 nm (Fig. 20). This is consistent with the redshift observed in a previous report.^142,143^ This shift is attributed to the π–π* transition of the graphitic C–C ring, indicating the restoration of the graphitic structure in rGO.

The redshift in the absorption peak towards higher wavelengths in rGO compared to GO confirms the successful reduction of oxygen-containing functional groups, leading to the restoration of the graphitic π-conjugated system. This restoration of the π-conjugated structure is a crucial aspect of rGO's improved electrical conductivity and other desirable properties. The absorption spectroscopy results provide valuable insights into the electronic structure and properties of GO and rGO, and they complement the information obtained from other characterization techniques, such as XRD and FTIR spectroscopy. Continued investigations in this area will deepen our understanding of the reduction process and its impact on the optical and electronic properties of graphene-based materials, opening new avenues for their application in diverse fields, including optoelectronics, sensors, and photovoltaics.^75,76^

#### 3.1.5. X-ray absorption spectroscopy

X-ray Absorption Spectroscopy (XAS) is a powerful analytical technique for studying MXenes, offering valuable insights into their structural and electronic properties. Although traditionally requiring synchrotron radiation sources, desktop systems with limited capabilities have made XAS more accessible. XAS encompasses two main methods: X-ray Absorption Near-Edge Structure (XANES) and Extended X-ray Absorption Fine Structure (EXAFS).

XANES provides information about the oxidation state of atoms in MXenes, while EXAFS reveals details about bond length and coordination number. These techniques have been extensively employed to investigate the electronic states of MXenes, elucidating the oxidation states of various transition metals. For instance, XANES spectra have revealed that Mo in Mo_2_CT_*x*_ exhibits an oxidation state similar to MoO_2_ (+4), while Nb_2_CT_*x*_ and Ti_3_C_2_T_*x*_ display oxidation states akin to NbC and TiC, respectively. Notably, the oxidation state of Ti in MXenes can vary depending on factors such as the number of transition metal layers and the outer layer composition, impacting the properties of ordered double transition metal MXene.^141,142^

XAS is often combined with *in situ* experiments to gain insights into how fundamental properties change during device operations. For example, XANES spectra were employed to monitor the oxidation state of Ti in Ti_3_C_2_T_*x*_ during cycling in an acidic electrolyte, revealing redox energy storage by Ti_3_C_2_T_*x*_. Similarly, changes in the oxidation state of V in V_2_CT_*x*_ during Na-ion battery cycling were investigated using XAS. The technique has also been employed to study the effect of intercalation, such as urea intercalation, on the oxidation state of titanium in Ti_3_C_2_T_*x*_. Furthermore, XAS spatial resolution has allowed researchers to observe variations in the oxidation state of Ti at the edges compared to the basal plane.

### 3.2. Microscopy techniques

#### 3.2.1. Atomic force microscopy (AFM)

##### 3.2.1.1. Graphene

**Fig. 21:**
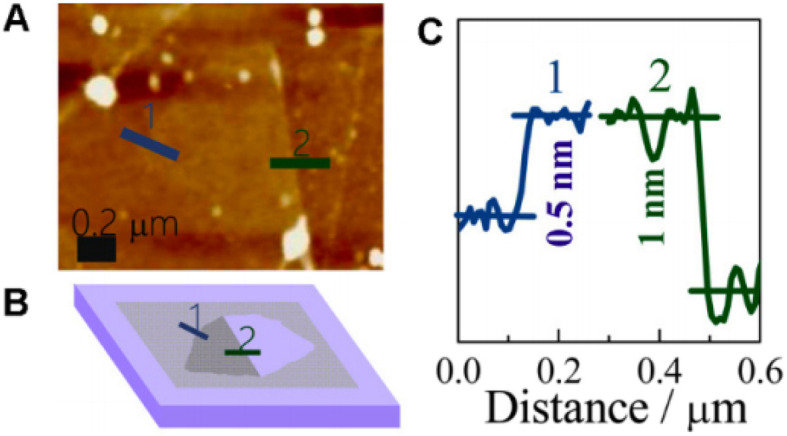
AFM measurement of folded FeN-MLG. (A) AFM image. (B) Bright spot morphology. (C) Height profile marked by the solid lines 1 and 2 reproduced from ref. 111 with permission from the American Chemical Society, *ACS Catalysis*, X.-D. Yang, Y. Zheng, J. Yang, *et al.*, 2017, **7**, 1, © 2017 American Chemical Society.

AFM is a prevalent method for measuring thickness and counting layer number of graphene-based materials. During a measurement, a sharp tip (5–10 nm) on a cantilever scans across the sample surface, collecting data on changes in vibration amplitude and frequency due to the sample's surface heterogeneity to create a topographic image.^145^ These three-dimensional images are used to determine the thickness of graphene films, and with a perfect graphene having a single atom thickness of 0.35 nm, the number of layers can be determined.^30^ AFM can also determine surface roughness, providing information on surface area and active area.^146^ Studied Pd–Fe/graphene catalysts produced through photocatalytic reduction, examining their performance for electrochemical oxidation–reduction of chlorophenols using AFM. The AFM image of the Pd_0.5_Fe_0.5_/graphene catalyst showed an irregular crystal block with an average thickness of 1.2 nm (Fig. 21).

##### 3.2.1.2. MXenes

Atomic Force Microscopy (AFM) is a widely used technique for analysing 2D materials, offering valuable information about their lateral dimensions and thickness. However, when determining the thickness of 2D monolayers using AFM, caution must be exercised due to potential influences from surface adsorbates, trapped interfacial molecules, and the imaging method employed. To achieve accurate measurements, it is often preferable to determine the height of the second layer instead of directly measuring the monolayer on the substrate. This approach has also been applied to MXenes.

For example, in the case of Ti_3_C_2_T_*x*_ MXene, the height of the second layer was measured to be 1.6 nm, while the MXene layer directly on the substrate appeared to have a height of up to 3.0 nm. This discrepancy resulted in an overestimation of the thickness compared to its nominal thickness of approximately 1 nm, as confirmed by high-resolution TEM and computational methods. As a result, cross-sectional TEM is considered a more reliable technique for accurately measuring the thickness of monolayers.^147,148^

It is worth noting that, to date, no scanning tunnelling microscopy (STM) studies have been conducted on MXenes. As research in this field continues to evolve, the use of advanced imaging techniques will likely contribute to a deeper understanding of MXenes' structural properties and their applications in various fields, including energy storage, catalysis, and electronics. The combination of complementary characterisation techniques, such as AFM, TEM, and STM, will play a crucial role in providing comprehensive insights into MXene materials and their potential for various technological advancements.

#### 3.2.2. Scanning electron microscopy and transmission electron microscopy

##### 3.2.2.1. Graphene

**Fig. 22:**
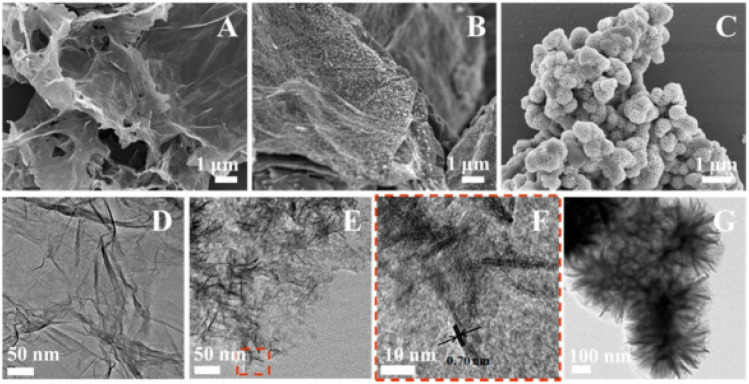
SEM and TEM images of graphene (A and D), graphene–MnO_2_ (B and E), and MnO_2_ samples (C and G); (F) HRTEM image of graphene–MnO_2_ reproduced from ref. 111 with permission from the American Chemical Society, *The Journal of Physical Chemistry C*, L. Lu, H. Tian, J. He, *et al.*, 2016, **120**, 41, © 2016 American Chemical Society.

SEM showed that graphene has a 2D planar structure with well-defined interconnected pores. This structure is believed to enhance adsorption and increase active sites for catalysis. Wrinkles and folds were seen, highlighting graphene's flexibility.^149^ TEM and HRTEM^150^ showed uniform dispersal of MnO_2_ nanoparticles in the shape of nanoneedles on the graphene surface, attributed to the graphene's 2D planar architecture.^151^ The nanoneedles also prevented aggregation to some extent. HRTEM revealed a *d*-spacing of 0.70 nm assigned to MnO_2_ (003), indicating its crystalline nature. The dispersion of MnO_2_ was due to graphene's 2D planar structure,^138^ while the nanoneedle-shaped MnO_2_ prevented graphene aggregation. HRTEM found a 0.70 nm *d*-spacing assigned to MnO_2_ (003), confirming its crystalline structure^152^ (Fig. 22).

##### 3.2.2.2. Graphene oxide and reduced graphene oxide

**Fig. 23:**
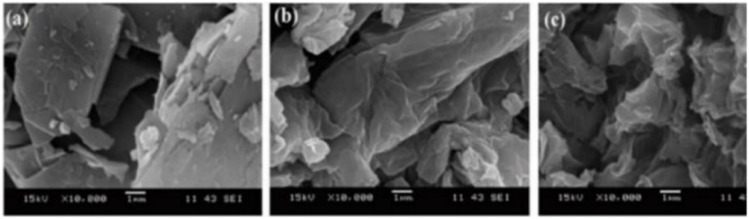
SEM images of (a) Graphene (b) GO and (c) rGO reproduced from ref. 153 P. Viprya, D. Kumar and S. Kowshik, *Eng. Proc.*, 2023, **59**, 84, © 2023. The authors (CC BY 4.0).

Scanning Electron Microscopy (SEM) was employed to examine the morphologies of graphene, GO, and rGO samples. The SEM micrographs obtained at a magnification of 10000× provided highly magnified images of the material's surface. In Fig. 23 the micrographs for graphene, GO, and rGO samples are displayed.

Fig. 23(a) illustrates the micrograph of graphene, revealing a platelet-like crystalline form of carbon. Moving on to Fig. 23(b), the SEM micrograph of GO shows the presence of wrinkled and layered flakes on the surface, indicating that the graphene layers were fully oxidized to GO. These distinctive flakes are a characteristic feature of GO.

In Fig. 23(c), the micrograph of rGO obtained after chemical reduction using hydrazine hydrate is displayed. The rGO surface exhibits crumpled thin sheets that accumulate to form a disordered structure. This transformation from the wrinkled and layered flakes of GO to the crumpled sheets of rGO signifies the successful reduction process.^154^

Remarkably, the morphologies of rGO in Fig. 23(c) closely resemble those reported in the previous work,^142^ despite their use of the modified Hummers' method, whereas in this study, the improved Hummers' method was employed. This similarity in morphology confirms the effectiveness of the reduction process in both methods.

The SEM observations provide essential visual evidence of the structural changes that occur during the oxidation and reduction processes, enabling a better understanding of the material's properties and behavior. These morphological insights play a crucial role in tailoring and optimizing the properties of graphene-based materials for various applications in fields such as nanoelectronics, composites, and energy storage devices. Further studies in this direction will undoubtedly contribute to the advancement and utilization of graphene-based materials in cutting-edge technologies.^155,156^

##### 3.2.2.3. MXenes

**Fig. 24:**
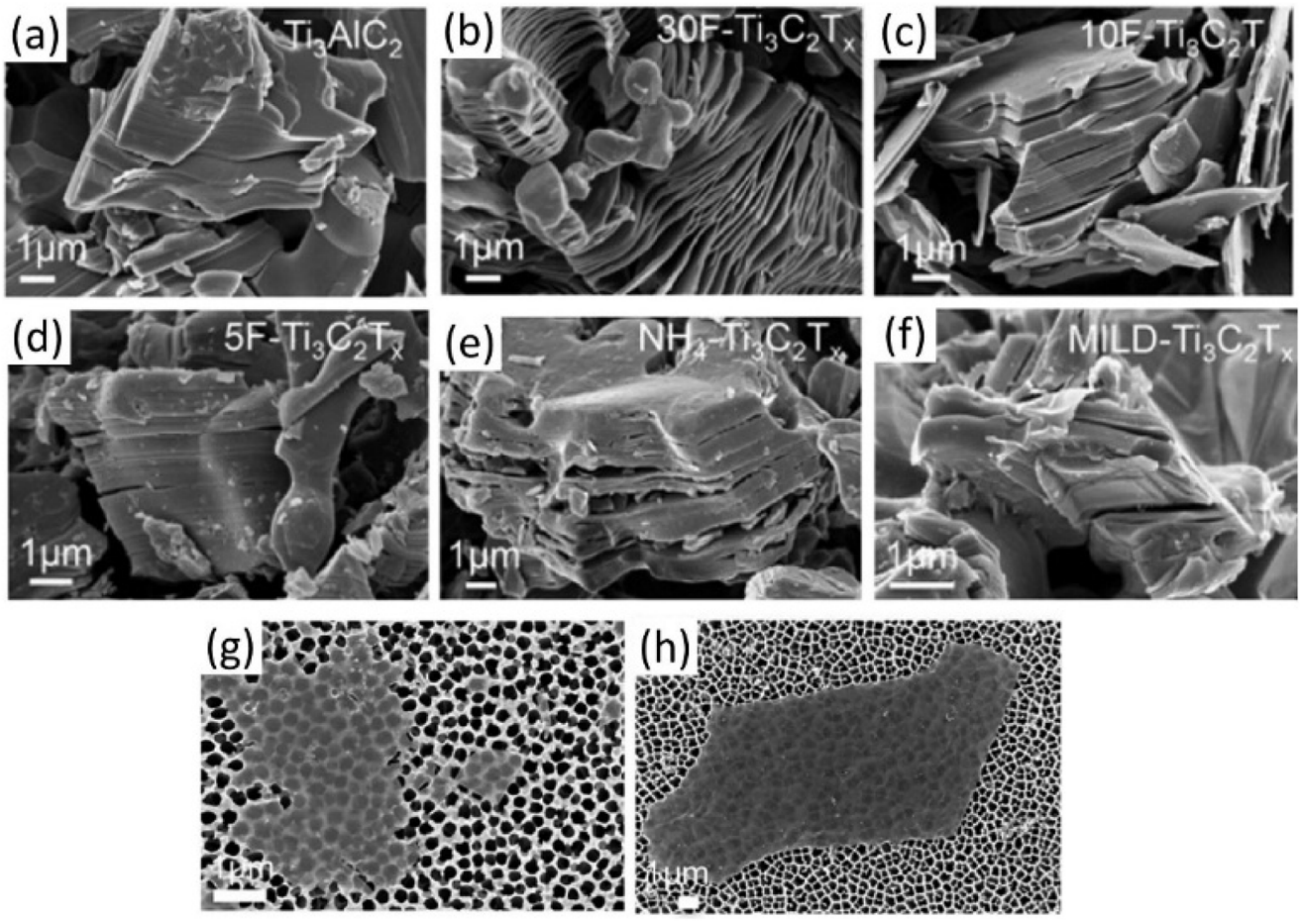
The SEM images depict: (a) Ti_3_AlC_2_ (MAX) powder displaying its compact layered structure; (b)–(d) multilayered Ti_3_C_2_T_*x*_ powders synthesized with 30 wt%, 10 wt%, and 5 wt% HF, respectively, with only the 30 wt% HF etching revealing an accordion-like morphology. (e) Multilayered NH_4_–Ti_3_C_2_T_*x*_ powder synthesized using ammonium hydrogen fluoride, and (f) MILD method (etched with LiF in HCl), both exhibiting minimal opening of MXene lamellas, akin to observations in 5F–Ti_3_C_2_T_*x*_. Additionally, (g) and (h) SEM images illustrate single MXene flakes on a porous alumina substrate, etched using 5 wt% HF and the MILD method, respectively reproduced from ref. 157 with permission from the American Chemical Society, *Chemistry of Materials*, M. Alhabeb, K. Maleski, B. Anasori, *et al.*, 2017, **29**, 18, © 2017 American Chemical Society.

Scanning electron microscopy (SEM) is a powerful technique widely used to visualize and confirm the formation of MXene structures. However, it's essential to acknowledge that not all etched MXene samples exhibit the characteristic “accordion” structure, which was initially observed in MXene synthesis using high-concentration HF. SEM images of Ti_3_C_2_T_*x*_ synthesized under different conditions (Fig. 24) demonstrate that as the HF concentration decreases, the accordion structure diminishes, and the multilayer MXene adopts a more typical MAX structure appearance. The presence or absence of the accordion structure is dependent on the kinetics of H_2_ production during HF treatment.

*In situ* HF formation methods, like MILD (mild etching of Al in LiF), prior to delamination, yield MXene structures similar to those produced with low-concentration HF. Additionally, SEM analysis can reveal the presence of oxide nanoparticles on the MXene surface due to severe etching and oxidation, which may not be detected by XRD but can be observed through techniques like Raman spectroscopy or X-ray photoelectron spectroscopy (XPS). Such oxide nanoparticles are more likely to be observed when etching Ti_3_AlC_2_ in high-concentration HF for extended periods.^124^

Post-delamination, it is crucial to examine the MXene flakes using SEM, where they are drop-cast onto substrates like porous anodized alumina membrane (Fig. 24(g and h)). This enables the observation of flake size, dispersion, defects, and edge quality. Rather than merely confirming synthesis through images of MXene flakes, it is more valuable to conduct a comprehensive analysis and compare the flakes with other studies, especially in the development of new synthesis methods or the synthesis of novel MXenes. Different flake sizes and shapes can lead to varying properties and suitability for diverse applications.^158^

##### 3.2.2.4. Molybdenum sulfide

FESEM and TEM were performed to study the effect of distinct synthesis condition on the surface morphologies of MoS_2_ materials, the molar ratio between S/Mo, the different hydrothermal temperature and time. All the samples were synthesized with in molar ratio of 1 : 1 and at hydrothermal temperature about 240 °C and time about 37 h, these possesses layer like structure with 0.5 to 1 µm diameter. When S/Mo ratios were 1 : 1 and 2 : 1, some MoS_2_ with nano-flower structure developed on wrapped monolayer. For 3 : 1 and 4 : 1 Molar ratios a matchless structure of nano sheet could be noticed and the nano-flower structure on monolayer MoS_2_ vanished.^101^

**Fig. 25:**
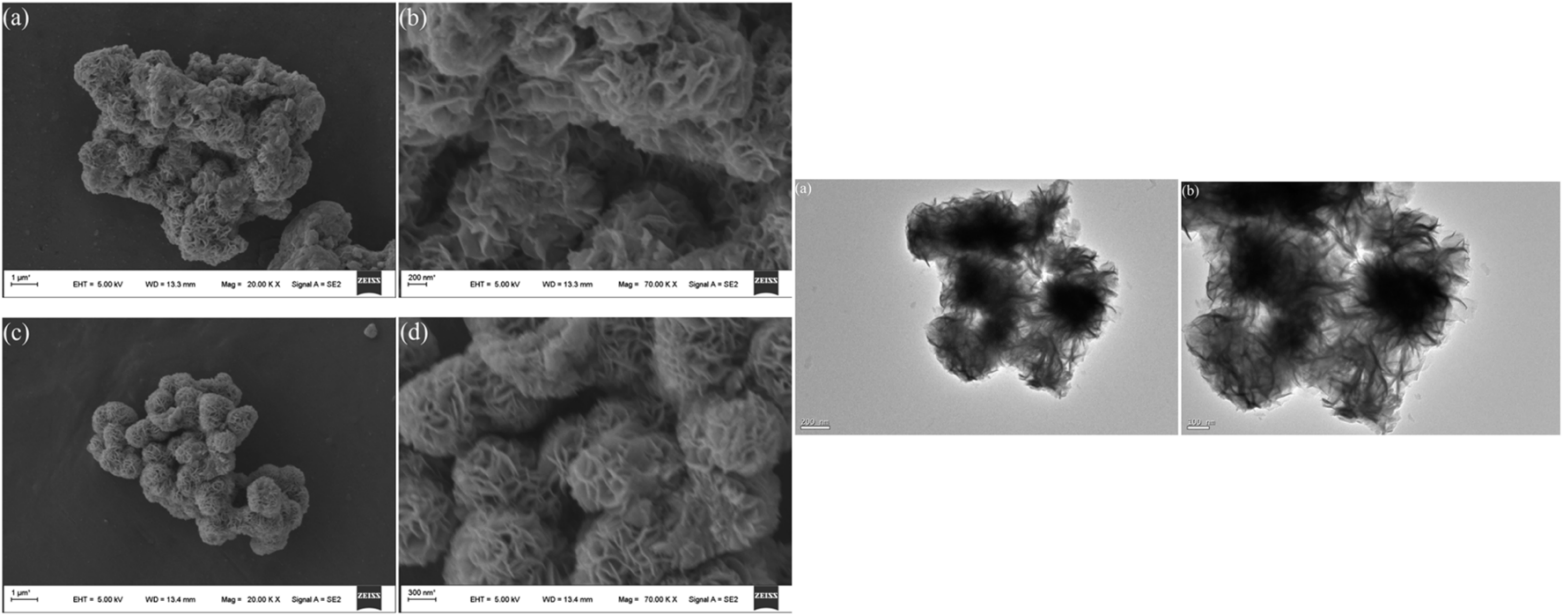
SEM images of MoS_2_ synthesized at different hydrothermal conditions: (a and b) MoS_2_-8 and (c and d) MoS_2_-16 showing coral-like and hierarchical flower-like morphologies. TEM images of MoS_2_-16 (right) reveal the internal structure of the assembled nanosheets reproduced from ref. 159 F. Wang, G. Li, J. Zheng, J. Ma, C. Yang and Q. Wang, *RSC Adv.*, 2018, **8**, 38945, © 2018. The authors, licensed under CC BY 3.0.

Fig. 25 illustrates the morphological evolution of MoS_2_ synthesized at a fixed molar ratio of 3 : 1 under different hydrothermal temperatures (150 °C, 180 °C, 210 °C, and 240 °C) and reaction times (25 h, 37 h, and 47 h). Fig. 25(a–c) show that with increasing hydrothermal time, the MoS_2_ fragments become smaller, and the samples MoS_2_-150-25, MoS_2_-150-37, and MoS_2_-150-47 tend to aggregate and form coral-like structures with a porous microstructure.

As shown in Fig. 25(d and e), hierarchical flower-like morphologies with diameters of approximately 0.5–1 µm are observed. These structures are composed of numerous petal-like units, where each petal consists of a large number of ultrathin nanosheets, indicating the strong tendency of MoS_2_ to self-assemble into nanoflower architectures. Furthermore, the size of the MoS_2_ nanoflowers increases as the hydrothermal time is extended from 25 h to 47 h.

When the hydrothermal temperature is increased to 210 °C, the MoS_2_ nanoflower structures begin to disintegrate and gradually transform into MoS_2_ nanosheets, as shown in Fig. 25(g–i). In addition, increasing the hydrothermal time from 25 h to 47 h results in the formation of larger nanosheets. At a hydrothermal temperature of 240 °C, the MoS_2_ nanosheets further increase in size, indicating enhanced crystal growth under higher temperature conditions.^159^

### 3.3. Other characterisations

#### 3.3.1. Pair distribution function analysis

PDF analysis is a valuable and powerful tool for investigating MXenes, providing essential insights into their structure at the atomic level. However, its application has been limited due to the requirement for large-scale facilities like synchrotrons or neutron sources. Nevertheless, this technique offers unparalleled benefits by determining the distances between all pairs of atoms in the MXene sample, enabling a comprehensive understanding of their structural details, including surface groups, element arrangement, and intercalants.

The combination of X-ray and neutron scattering data in PDF analysis allows researchers to delve deeply into MXene structures, even revealing the proportions of surface groups like OH and O. Despite the relatively few studies utilizing PDF on MXenes, they have yielded substantial information, such as the characterization of surface groups, the impact of etching on MXene structure, and the identification of M and X element locations in complex MXenes. Moreover, PDF has been instrumental in studying transformations from carbide to nitride MXenes and precisely determining the nature and location of intercalants between MXene layers.

Additionally, PDF analysis serves to validate predictions made by molecular dynamics or density functional theory, corroborating surface terminations or the presence of intercalated ions with water molecules. While not yet widely employed in MXene research, its potential for directly studying structural features, which are often accessible only through computational methods, is increasingly gaining recognition among research groups. Emphasizing and exploring the capabilities of PDF analysis in the MXene field could lead to groundbreaking discoveries and a deeper understanding of these fascinating 2D materials.^135,160,161^

#### 3.3.2. Brunauer–Emmett–Teller analysis

Measurements of BET surface area and pore size distributions from nitrogen physisorption isotherm analysis using BET models along with BJH or DFT methods are critical to assess ion access and transport kinetics in 2D electrodes being considered for use in battery or supercapacitor applications. Typically, graphene-derived materials (such as rGO or GNP) have high BET surface areas (typically between 300–800 m^2^ g; up to approximately 670 m^2^ g^−1^ for highly exfoliated forms) due to their atomically-thin sheet structure and large micropore/mesopore network facilitating electric double layer capacitance. Conversely, MXene film samples (for example, Ti_3_C_2_T_*x*_) generally display low BET surface area values (20–200 m^2^ g^−1^); Multilayered samples produce BET values of 6–18 m^2^ g^−1^ while delaminated flake samples produce BET values of 60–100 m^2^ g^−1^, despite theoretically high surface areas (over 1000 m^2^ g^−1^ per single layer).^162,163^

Although MXenes have lower BET areas compared to graphene, they exhibit higher volumetric capacitances due to pseudocapacitive intercalation of ions into the interlayer space instead of being reliant on surface adsorption of ions and thus outperform graphene when constrained to small device dimensions. Thus, optimizing the degree of delamination of MXene films and developing porous structure through engineering can effectively balance gravimetric and voltammetric performance of electrodes with practical electrode designs.

### 3.4. Summary of electrochemical performance of 2D materials

Tables 3 and 4 summarise the electrochemical performance of representative 2D material-based electrodes in supercapacitor and battery systems. As shown in Table 3, hybrid architectures incorporating rGO, transition metal oxides/sulfides, and heterostructured composites generally exhibit enhanced specific capacitance (>1000 F g^−1^ in several cases) and improved cycling stability due to synergistic redox activity and improved electron transport pathways. Similarly, Table 4 highlights that integrating 2D materials with high-capacity active phases such as Si, Li-rich NMC, and composite carbon matrices significantly improves reversible capacity and structural stability compared to conventional electrodes. These comparisons underscore the critical role of interface engineering and conductive network formation in achieving high-performance energy storage devices.

**Table 3:** Supercapacitor performance of 2D materials

Material	Electrolyte	Current density	Capacitance/capacity	Cycle life	Year	Ref.
TABQ-MWCNTs	1 M H_2_SO_4_	1 A g^−1^	463 F g^−1^	6000 cycles (76.8% retention)	2023	164
ZnO@Ni_3_S_2_ core–shell nanorods	1 M KOH	2 A g^−1^	1529 F g^−1^	2000 cycles (42% retention)	2025	165
MnO_2_ on 3D spongy NF	Na_2_SO_4_	1 A g^−1^	469 F g^−1^	5000 cycles (90% retention)	2025	165
ZSR NCs (ZnO/SnO_2_:rGO)	1 M Na_2_SO_4_ + RAE	1 A g^−1^	3238 F g^−1^	5000 cycles (91.54% retention)	2025	165
rGO-AHQDME	AN/EMIMBF_4_ (1 : 1)	1 A g^−1^	338 F g^−1^	10000 cycles (91% retention)	2023	164
NiO–Mn_2_O_3_@rGO	3 M KOH	1 A g^−1^	442 F g^−1^	500 cycles (91% retention)	2025	165
Co_3_O_4_/VS_4_/rGO-SDBS@NF	2 M KOH	1 A g^−1^	1250 F g^−1^	5000 cycles (97.4% retention)	2025	165
NiO–Fe–CNT	6 M KOH	1 A g^−1^	1360 F g^−1^	5000 cycles (92% retention)	2023	166
NPCs-2-700 (N-doped PC)	6 M KOH	1 A g^−1^	350 F g^−1^	5000 cycles (92% retention)	2019	165
AC-PPD-PBQ	6 M KOH	2 A g^−1^	144 F g^−1^	5000 cycles (84.4% retention)	2023	164

**Table 4:** Battery performance of 2D materials

Material (cathode//anode)	Electrolyte	Current density	Capacity	Cycle life	Year	Ref.
Fe^3+^-NCM871	Standard Li-ion	0.1 C	207.5 mAh g^−1^	Good cycle stability	2024	167
LFP (LiFePO_4_)	1 M LiPF_6_	0.1–0.5 C	>150 mAh g^−1^	3000–6000+ cycles (90%+ retention)	2024	168
LMR-NMC (Li-rich)	Organic electrolyte	Variable	280 mAh g^−1^	Lower than NMC811	2023	169
Graphite anode (pure)	1 M LiPF_6_ in EC:DMC	0.1 C	372 mAh g^−1^	500+ cycles	2019	170 and 171
Si–C composite (GSCC)	Standard Li-ion	0.2 A g^−1^	1100.6 mAh g^−1^	73% retention (100+ cycles)	2021	172
PAC–Si composite (porous artificial carbon–silicon)	Standard Li-ion	0.1 C	∼1000+ mAh g^−1^ (estimated)	Enhanced cycle life	2024	173
Si nanotubes (SiNTs)	Standard Li-ion	1 C	∼400–500 mAh g^−1^	89% retention @ 200 cycles	2025	174
All-solid-state (ceramic, Li–I_2_)	Solid electrolyte (ceramic separator)	C/3	Confined dissolution strategy	9000+ cycles (84.1% retention)	2025	175
NCM/Li half-cell (high-voltage 4.6 V)	LiPF_6_ organic	C/20 (45 °C)	180–190 mAh g^−1^	Limited (reversible capacity loss: 8.4 mAh g^−1^)	2019	176

## 4. Energy storage mechanisms

The field of energy storage has seen remarkable advancements with the emergence of 2D materials. These materials possess unique properties and surface reactivity that make them promising candidates for energy storage applications. Among the various energy storage mechanisms explored in 2D materials, three prominent ones stand out: intercalation, adsorption, and conversion reactions. Each mechanism offers distinct advantages and limitations, providing a diverse range of options for designing advanced energy storage devices.

### 4.1. Intercalation

Intercalation is a process where guest ions or molecules are inserted between the layers of a 2D material's lattice. This mechanism is commonly observed in TMDs and other layered materials. During charging or discharging, ions or molecules (*e.g.*, Li^+^, Na^+^, K^+^) are inserted or extracted between the atomic layers of the 2D material. The intercalation process allows for efficient ion diffusion and provides high charge storage capacity, making it suitable for rechargeable batteries. For example, GO and its reduced form, rGO, can undergo intercalation of ions or molecules between their layered structures. For example, lithium ions can intercalate into the layers of GO or rGO,^177^ enabling their use as anodes in lithium-ion batteries. The large surface area and high electrical conductivity of graphene-based materials make them ideal candidates for intercalation-based energy storage systems.^151^

Intercalation allows for the insertion of a significant number of guest ions or molecules between the layers of 2D materials, leading to high charge storage capacity and energy density. The layered structure of 2D materials provides pathways for rapid ion diffusion, enabling fast charge and discharge rates. Intercalation reactions are generally reversible, allowing for repeated charging and discharging cycles without significant capacity loss.^52^

Some 2D materials may have limited redox activity, leading to lower specific capacities than other energy storage mechanisms. Repeated ion intercalation and deintercalation can cause structural degradation and affect the long-term cycling stability of the material.^178^

### 4.2. Adsorption

Adsorption is a mechanism in which gas molecules, ions, or molecules from an electrolyte are adsorbed onto the surface of 2D materials. This process involves weak interactions between the adsorbates and the material's surface, such as van der Waals forces or physisorption. The high surface area of 2D materials facilitates efficient gas or ion adsorption, leading to enhanced energy storage performance. MXenes, such as Ti_3_C_2_T_*x*_, show excellent energy storage performance through the adsorption of ions on their 2D surfaces.^179^ They possess functional groups on their surfaces that facilitate ion adsorption and desorption. MXenes have been explored as electrode materials in supercapacitors, exhibiting high specific capacitance and rapid charge/discharge rates due to their adsorption-based energy storage mechanism.

2D materials typically possess a large surface area, providing ample sites for gas or ion adsorption and leading to high energy storage capacities. Adsorption processes involve weak interactions, enabling fast charge and discharge rates. Adsorption is a reversible mechanism, allowing for repeated adsorption and desorption cycles.^180^ In some cases, the adsorption sites on the surface of the 2D material may become saturated, limiting the amount of ions or gas that can be stored. Adsorption processes can be affected by changes in temperature, humidity, or the composition of the surrounding atmosphere, leading to variations in performance.

### 4.3. Conversion reactions

Conversion reactions involve chemical transformations of the 2D material during energy storage. In these reactions, the structure of the material changes reversibly during charging and discharging. This mechanism often leads to higher energy densities but can suffer from limited cycling stability. Transition metal oxides, such as MoO_3_ and MnO_2_,^181^ are examples of 2D materials that undergo conversion reactions in lithium-ion batteries. During charging, the metal oxide undergoes a conversion reaction with Li+ ions, resulting in the formation of new compounds. In subsequent discharging, the new compounds are converted back to the original metal oxide structure.^182^

Conversion reactions can lead to higher energy densities compared to intercalation or adsorption mechanisms due to the formation of new compounds. Conversion reactions often involve multiple redox processes, providing multiple charge storage mechanisms, which can enhance energy storage capabilities.^183^

Some conversion reactions may suffer from incomplete reversibility, leading to capacity fading and reduced cycling stability. Conversion reactions can involve complex chemical transformations, leading to slow reaction kinetics and reduced rate capability. During conversion reactions, 2D materials may undergo significant volume changes, leading to mechanical stress and structural degradation.

### 4.4. Pseudocapacitance

Pseudocapacitance is a unique energy storage mechanism utilized in 2D materials, characterized by reversible redox reactions occurring at the electrode–electrolyte interface. Unlike the conventional electrical double-layer capacitance observed in supercapacitors, pseudocapacitance involves faradaic reactions, where charge is stored through the reversible transfer of electrons between the electrode and the electrolyte. This redox process leads to the formation of a double layer that contributes significantly to the overall charge storage capacity. For example, transition metal oxides and hydroxides, such as ruthenium oxide (RuO_2_), manganese oxide (MnO_2_),^184^ and nickel hydroxide (Ni(OH)_2_), are commonly used as pseudocapacitive materials in energy storage devices. During charging, these materials undergo redox reactions, where metal cations change their oxidation states, accompanied by the reversible adsorption and desorption of ions from the electrolyte. These redox processes allow for efficient and reversible charge storage, making pseudocapacitors promising candidates for high-performance energy storage systems.^185^

Pseudocapacitors can exhibit higher energy density than electrical double-layer capacitors (EDLCs) due to the additional redox reactions, allowing for more efficient charge storage. Pseudocapacitive materials can provide rapid charge and discharge rates, making them suitable for applications requiring quick energy transfer. Many pseudocapacitive materials exhibit excellent cycling stability, resulting in long-lasting energy storage devices with minimal degradation over numerous charge–discharge cycles. Pseudocapacitors can deliver high power output, making them ideal for high-power applications.

The specific capacitance of pseudocapacitive materials may not be as high as some intercalation-based systems, limiting the total amount of charge they can store. Pseudocapacitive materials often have limited voltage windows compared to intercalation-based systems, which can impact their overall energy storage capacity. The performance of pseudocapacitive materials depends on the reversibility of the redox reactions. In some cases, irreversible redox reactions may lead to capacity fading over time.

## 5. Recent advances in 2D materials for energy storage

Recent years have witnessed remarkable progress in the development of 2D materials for energy storage applications. These atomically thin materials offer unique properties and surface reactivity that make them ideal candidates for various energy storage mechanisms. From supercapacitors to batteries and photocatalytic devices, 2D materials have shown great potential in revolutionizing energy storage technologies. The following highlights some of the latest advancements in this dynamic field, focusing on materials like MXenes, transition metal dichalcogenides, and graphene derivatives, as well as emerging concepts like van der Waals heterostructures and pseudocapacitive behavior. With continuous research efforts driving scalable synthesis methods and large-scale production, 2D materials are poised to play a pivotal role in shaping the future of sustainable and efficient energy storage.

### 5.1. Batteries

#### 5.1.1. 2D transition metal dichalcogenides (TMDs) for batteries

2D TMDs for batteries: TMDs, such as MoS_2_ and WS_2_, have gained attention as electrode materials in lithium-ion batteries. Their ability to undergo conversion reactions during lithium intercalation offers high specific capacities and energy densities.^124^ Researchers are exploring various strategies to improve their rate capabilities and cyclability.

Research conducted by Chaoui^186^ demonstrates that MoS_2_ and WSe_2_ monolayer exhibit potential as a new generation of anode materials for lithium (Li), sodium (Na) and potassium (K)-ion batteries; and have demonstrated a very low diffusion energy barrier of 0.24 eV for Li on MoS_2_ which is expected to allow for rapid ion transport and very high theoretical capacities in composite material in excess of 1000 mAh g^−1^. A 2024 study of MoS_2_, WS_2_ and MoWS_2_ flakes shows superior Na-ion battery performance for MoS_2_ with an initial charge capacity of 1056 mAh g^−1^ and a coulombic efficiency of 46% due to decreased electrical resistance caused by the smaller size of the alkali ions and the synergistic effects that result from alloying and enhance cyclic stability.^187^

#### 5.1.2. Graphene and its derivatives for energy storage

Graphene and its derivatives for energy storage: graphene and its derivatives, such as graphene oxide and reduced graphene oxide, continue to be extensively studied for various energy storage devices.^188^ In the study by Mishra,^189^ graphene oxide–lithium hybrids pioneered flexible, high-capacity lithium-ion batteries with capacities up to 900 mAh g^−1^ and 95% retention after 500 cycles, leveraging oxygen functional groups for enhanced Li^+^ binding and structural stability in solid-state electrolytes.

#### 5.1.3. Advancements in electrode materials

MXenes: MXenes, derived from MAX phases, have emerged as promising electrode materials for energy storage due to their high electrical conductivity and large surface area. Their 2D nature allows for facile ion diffusion and high charge storage capacity. MXenes have been utilized in supercapacitors,^179^ lithium-ion batteries,^172^ and sodium-ion batteries, exhibiting impressive electrochemical performance and long-term stability.

Transition metal dichalcogenides (TMDs): TMDs, such as MoS_2_ and WS_2_, have gained attention as electrode materials for batteries due to their unique layered structure and tunable electronic properties.^190^ Their ability to accommodate ion intercalation and surface redox reactions enables high specific capacitance and capacity in energy storage devices.

Zhou^191^ demonstrated that multiple transition metal MXenes enhance electronic conductivity and lattice distortion in composites, enabling uniform ion deposition and high-rate performance in supercapacitors with improved cycling stability due to optimized surface chemistry. Roy^192^ showed MoS_2_ flakes achieving 1056 mAh g^−1^ initial capacity and 71% retention in Na-ion batteries, outperforming WS_2_ due to lower resistance from smaller ions and alloying in MoWS_2_ for enhanced cyclic stability.

#### 5.1.4. Advancements in electrolytes

Solid-state electrolytes: solid-state electrolytes have emerged as a potential alternative to conventional liquid electrolytes, offering higher thermal stability and reduced safety concerns. 2D materials, such as boron nitride, have been incorporated into solid-state electrolytes to enhance ion conductivity and prevent dendrite formation, enabling safer and more efficient battery operation.^193^

Ionic liquids: ionic liquids are non-volatile and thermally stable electrolytes that have shown promise in enhancing the performance of energy storage devices. Incorporating 2D materials, like graphene,^167^ into ionic liquids can further improve ion mobility and reduce resistance, leading to higher power densities and longer cycle life in batteries.^86,87^

Sarfraz^194^ reviewed current advancements in solid inorganic electrolytes for all-solid-state lithium batteries; Boron Nitride Nanosheet (BNNS) solid electrolytes were noted to have a significant increase in ionic conductivity of 10^−3^ S cm^−1^ and suppression of dendrites during cycling by having an even Li^+^ flux distribution and mechanical reinforcement at interfaces. Wang^195^ applied Machine Learning (ML) to select from ionic liquid–solid hybrid electrolytes with graphene filler materials; ML identified specific composition(s) that increased ion mobility to 10^−2^ S cm^−1^ and reduced interface resistances by 50% and support high power densities in lithium metal batteries.

#### 5.1.5. Advancements in overall battery performance using 2D materials

Advancements in overall battery performance using 2D materials, with their large surface area and unique electronic properties, enable high-capacity electrode design, leading to higher energy-density batteries. These materials can store a larger amount of charge per unit mass or volume, offering longer-lasting and more energy-efficient batteries. Using 2D materials in electrodes and electrolytes facilitates faster ion diffusion and charge transfer kinetics. This results in batteries that can be charged and discharged at higher rates, enabling rapid charging capabilities and shorter device response times. The stability and mechanical robustness of 2D materials contribute to prolonged cycle life in energy storage devices. Reduced electrode degradation and electrolyte decomposition enhance battery performance and longer service life. 2D materials, particularly graphene and its derivatives, are abundant, lightweight, and environmentally friendly. The utilisation of these materials in energy storage devices promotes sustainable battery technologies with reduced reliance on rare and toxic elements.

### 5.2. Supercapacitors

#### 5.2.1. Supercapacitors and capacitive energy storage

Supercapacitors, also known as ultracapacitors or electrochemical capacitors, are energy storage devices that can store and release a large amount of electrical energy quickly. They operate based on the principle of “capacitance” and are distinct from batteries and conventional capacitors due to their unique energy storage mechanism. Capacitance is a measure of how much charge a capacitor can store per unit of voltage.

In supercapacitors, the electrodes are typically made of porous materials with high surface areas, such as activated carbon, carbon nanotubes, or various 2D materials like graphene and MXenes.^196^ When a voltage is applied across the electrodes immersed in an electrolyte, ions in the electrolyte are adsorbed onto the surface of the electrode, forming a layer of charge known as the “electric double layer.” This double layer comprises two regions: the “Helmholtz layer,” which contains specifically adsorbed ions, and the “diffuse layer,” which contains ions loosely associated with the electrode surface. The adsorption of ions at the electrode–electrolyte interface leads to a significant increase in the effective capacitance of the supercapacitor, resulting in high charge storage capacity. As a result, supercapacitors can deliver large amounts of charge and discharge rapidly, making them ideal for applications that require high power delivery, such as regenerative braking in electric vehicles and powering electronic devices during peak demand.

Due to their unique properties and large surface areas, 2D materials have emerged as promising candidates for various applications in supercapacitors and capacitive energy storage devices. These materials offer distinct advantages, such as high electrical conductivity, large surface-to-volume ratios, and tunable properties, making them ideal for enhancing the performance of energy storage devices. Some key applications of 2D materials in supercapacitors and capacitive energy storage devices include:

• Electrode materials: 2D materials, particularly graphene and MXenes, have been extensively studied as electrode materials in supercapacitors.^197^ Their high electrical conductivity and large specific surface areas allow for efficient charge storage and rapid charge–discharge rates. Graphene-based electrodes have demonstrated excellent capacitance, while MXenes have shown pseudocapacitive behavior, enhancing the overall energy storage capacity.

• Electrolytes: ionic liquids^198^ and other advanced electrolytes containing 2D materials are being explored to improve the performance of supercapacitors. These electrolytes can enhance the charge transfer kinetics, increase the capacitance, and extend the cycle life of supercapacitors.^193^

• Flexible and wearable devices: 2D materials' mechanical flexibility and lightweight nature make them suitable for integrating into flexible and wearable energy storage devices. Graphene and other 2D materials can be incorporated into flexible electrodes or supercapacitor films, enabling energy storage solutions for wearable electronics and flexible electronics.^199^

• High-power applications: 2D materials' ability to deliver high power and rapid charge–discharge rates makes them ideal for applications that require quick bursts of energy, such as regenerative braking in electric vehicles, power electronics, and energy harvesting systems.^200^

• Hybrid supercapacitors: 2D materials are often used in hybrid supercapacitors that combine the advantages of batteries and supercapacitors.^201^ These hybrid devices can provide high energy density, rapid charge–discharge rates, and long cycle life, making them suitable for various energy storage applications.

• Energy storage for IoT and portable electronics: 2D materials offer the potential to develop lightweight, compact, and efficient energy storage solutions for the Internet of Things (IoT) devices and portable electronics, reducing the reliance on traditional batteries.^202^

• High-performance electrodes for capacitive deionization: capacitive deionization (CDI) is an energy-efficient water desalination technique that utilizes electrochemical principles. 2D materials' high surface area and charge storage capacity make them excellent candidates for CDI electrodes, enabling efficient and environmentally friendly desalination processes.^203^

• Supercapacitor-based energy storage systems: integrating supercapacitors with other energy storage systems, such as batteries and fuel cells, can create hybrid energy storage systems that optimize energy management and provide a reliable and sustainable power supply.^204^

The Nazarpour-Fard^205^ demonstrated an asymmetric supercapacitor that was composed of N-doped graphene/MXene electrodes in a neutral KNO_3_ electrolyte. The supercapacitor demonstrated a capacitance retention of 90.4% after 25 000 cycles, which was attributed to increased pseudocapacitance and conductivity. Zhao^206^ developed flexible all-in-one MXene supercapacitors *via* scalable blade coating, providing a high areal capacitance of 77.25 mF cm^−2^ as well as a high percentage retention (68%) at high scan rates; thus allowing them to be suitable for wearable devices. Zhang created coaxial fibrous supercapacitors *via* one-step wet-spinning of N-doped MXene-carbon nanosheets with Ag deposition. They were able to demonstrate a high energy density of 402.8 µWh cm^−2^ as well as good bending stability. Jimenez^207^ built an all-solid-state supercapacitor that utilized a dry multilayered PDDA/graphene oxide electrolyte assembled by layer-by-layer stacking. They were able to build supercapacitors with preserved capacitive behavior up to 20 layers through the use of both EDL and pseudocapacitance for rapid discharging. Momin^208^ created composite electrodes of graphene amine/MXene that exhibited superior electrochemical performance with a high specific capacitance due to synergistic interfacial effects between the two materials in supercapacitors.

2D materials have shown remarkable improvements in energy density, power density, and cycling stability when utilized in energy storage devices, particularly supercapacitors. These enhancements are attributed to their unique properties and surface reactivity, which enable efficient charge storage and rapid charge–discharge rates.

#### 5.2.2. MXenes for supercapacitors

MXenes for supercapacitors: MXenes, a family of 2D transition metal carbides, nitrides, and carbonitrides, have shown great promise in supercapacitor applications. Their unique layered structure and high surface area enable efficient ion adsorption and fast charge/discharge rates. Recent studies have focused on optimizing the surface terminations and introducing functional groups to enhance their capacitance and cycling stability.

MXene-based supercapacitors with ultrahigh energy density: In recent years, researchers have demonstrated the exceptional energy storage capabilities of MXenes in supercapacitor applications. For example, a breakthrough study reported a 2D MXene-based supercapacitor that achieved an ultrahigh energy density of over 350 Wh kg^−1^, which surpasses the energy density of conventional electrolytic capacitors and even approaches the energy density of some lithium-ion batteries. This advancement is attributed to the unique combination of high surface area, fast ion diffusion, and pseudocapacitive behaviour in MXenes.^209^ The potential impact of such breakthroughs lies in revolutionising energy storage technologies, enabling more compact and efficient energy storage devices with faster charge–discharge rates and longer cycle life.^210,211^

Ashram Alam^212^ developed a cathode/anode system by embedding pseudocapacitive FeZnS and MnZnS nanoparticles within Ti_3_C_2_T_*x*_ (MXene) resulting in a high specific capacitance of 366.4 F g^−1^ and an ultra-high energy density of 130.27 Wh kg^−1^ at 800 W kg^−1^ while maintaining over 6000 cycles. The Ghosh group^213^ synthesized an asymmetric ZCO–WO_3_@MXene nanoflower//MXene-rGO supercapacitor that demonstrated a 28 Wh kg^−1^ energy density at a 578 W kg^−1^ power density in pouch cells and maintained 93% of its original capacitance after 5000 cycles through improved charge-transfer kinetics and mechanical stability.

### 5.3. Other energy storage

Apart from batteries and supercapacitors, 2D materials are also finding applications in other energy storage technologies, such as fuel cells, hydrogen storage, and solar cells.

#### 5.3.1. Fuel cells

Fuel cells are electrochemical devices that convert the chemical energy of a fuel (*e.g.*, hydrogen) and an oxidant (*e.g.*, oxygen) directly into electrical energy.^214–216^ 2D materials have shown promise in various components of fuel cells (Table 5):

**Table 5:** Key components, materials, and advantages of PEM fuel cell electrodes

Component	Materials	Properties/advantages
Catalyst support	Platinum-based catalysts, 2D materials (*e.g.*, graphene, TMDs)	Increases active surface area and improves catalytic performance; graphene and TMDs are alternatives to expensive and scarce platinum catalysts
Gas diffusion layers (GDLs)	Graphene-based materials	High electrical conductivity and gas permeability, facilitate reactant transport and water management
Proton exchange membranes (PEMs)	2D materials (*e.g.*, graphene oxide, MXenes)	High proton conductivity, thermal stability, mechanical flexibility

Advantages: 2D materials provide a large surface area for catalytic reactions, enhancing the efficiency of fuel cell electrodes. Certain 2D materials, such as TMDs and graphene-based composites, exhibit excellent catalytic properties, reducing the reliance on expensive and scarce platinum-based catalysts. Graphene and MXenes, being mechanically flexible, can improve the durability and stability of proton exchange membranes (PEMs) in fuel cells. 2D materials like graphene oxide and MXenes can facilitate efficient proton transport in PEMs, improving the overall performance of the fuel cell.

Limitations: some 2D materials may have lower electrical conductivity compared to traditional carbon-based materials, affecting their performance in gas diffusion layers. The large-scale production of high-quality 2D materials can be challenging, leading to potential cost and scalability issues.

#### 5.3.2. Hydrogen storage

Hydrogen is a clean and efficient energy carrier, but its storage remains a significant challenge. 2D materials offer potential solutions for hydrogen storages^217,218^ (Table 6).

**Table 6:** 2D materials in hydrogen storage processes

Process	Materials	Mechanism/property
Physisorption	Graphene, other 2D materials	Hydrogen molecules'physisorbed on the surface and weak van der Waals interactions, Provide reversible and safe hydrogen storage
Surface functionalization	2D materials (*e.g.*, graphene)	The surface can be functionalised with specific groups, enhances hydrogen adsorption and desorption properties

Advantages: 2D materials provide a large surface area for hydrogen adsorption, maximising the hydrogen storage capacity. Physisorption-based hydrogen storage on 2D materials offers a reversible and safe hydrogen storage and release method. The properties of 2D materials can be tailored through functionalisation, optimising their hydrogen storage capacity.

Limitations: physisorption-based hydrogen storage has relatively low storage capacities compared to other storage methods, limiting the range of practical applications. The hydrogen desorption rate from 2D materials can be slow, hindering rapid cycling and recharge in hydrogen storage systems.

#### 5.3.3. Solar cells

2D materials have garnered interest in the field of solar cells due to their unique optoelectronic properties and potential for low-cost and flexible devices^219,220^ (Table 7).

**Table 7:** 2D materials in solar cell components

Solar cell component	Materials	Properties/advantages
Photovoltaic absorbers	TMDs, perovskite-like 2D materials	Tunable bandgaps, strong light absorption capabilities, suitable as light-absorbing layers in solar cells
Transparent conductive electrodes	Graphene, other 2D materials	Transparent conductive electrodes, improve light transmission, enhance charge collection efficiency in solar cells
Electron transport layers	2D materials	Efficient electron transport layers, facilitate charge transfer and enhances the overall efficiency of solar cells

Advantages: TMDs and perovskite-like 2D materials offer tunable bandgaps, enabling efficient light absorption over a wide range of solar spectrum. Graphene and other 2D materials can be produced relatively cheaply, making them attractive for low-cost and flexible solar cell applications. Graphene-based transparent conductive electrodes exhibit high electrical conductivity, improving charge collection efficiency in solar cells.

Limitations: some 2D materials may have limited light absorption in certain regions of the solar spectrum, reducing overall solar cell efficiency. The long-term stability and degradation of 2D materials in solar cells need to be addressed to ensure the reliability and longevity of the devices.

#### 5.3.4. Supercapacitors in hybrid energy storage

In addition to standalone supercapacitors, 2D materials are being integrated into hybrid energy storage systems^207,221^ (Table 8).

**Table 8:** MXenes and 2D materials in hybrid energy storage systems

Energy storage approach	2D materials used (*e.g.*, MXenes)	Features/advantages
Combining batteries and supercapacitors	2D materials in battery electrodes (anodes/cathodes) and supercapacitor electrodes	Improved energy density, improved power density, hybrid system benefits from both battery and supercapacitor characteristics
Battery-supercapacitor hybrid devices	Some 2D materials (*e.g.*, MXenes)	Exhibit battery-like intercalation behavior, possess capacitive properties, enables development of hybrid energy storage devices with combined features of batteries and supercapacitors

Advantages: 2D materials provide a large surface area for charge storage, contributing to the high capacitance of supercapacitor electrodes. The pseudo capacitance behaviour of some 2D materials enables rapid redox reactions, leading to fast charge–discharge rates in supercapacitors. Combining battery-like intercalation behaviour and capacitive properties in hybrid devices enhances the overall energy density.

Limitations: while hybrid energy storage devices improve energy density compared to traditional supercapacitors, they may still have lower energy density than batteries. The cycle life and long-term stability of 2D materials in hybrid supercapacitors need further investigation to ensure extended device lifetimes.

#### 5.3.5. 2D metal chalcogenides for photocatalytic water splitting

2D metal chalcogenides for photocatalytic water splitting: 2D metal chalcogenides, such as MoS_2_ and WS_2_, have shown promise as photocatalysts for water splitting.^191,222^ They exhibit excellent catalytic activity for the hydrogen evolution reaction under light irradiation, making them potential candidates for renewable energy conversion and storage.

##### 5.3.5.1. Van der Waals heterostructures for energy storage

Van der Waals Heterostructures for energy storage: researchers are exploring van der Waals heterostructures, which involve stacking different 2D materials layer by layer, to create novel energy storage devices.^223^ These heterostructures offer tunable electronic properties and unique charge transport pathways, leading to improved energy storage performance.

##### 5.3.5.2. Hydrogen storage

Hydrogen storage on 2D materials with improved desorption kinetics: hydrogen storage remains a critical challenge for fuel cell and hydrogen-powered vehicle technologies. Researchers have made significant progress in enhancing the hydrogen desorption kinetics on 2D materials. By engineering the surface chemistry and interlayer spacing of graphene and other 2D materials, hydrogen desorption rates have been accelerated, improving the practicality and efficiency of hydrogen storage systems. This breakthrough can potentially revolutionise the hydrogen economy, enabling safer and more efficient hydrogen storage for various applications.^224^

##### 5.3.5.3. Van der Waals heterostructures for energy conversion

Van der Waals heterostructures for energy conversion: Van der Waals heterostructures, which involve stacking different 2D materials with weak interlayer interactions, have emerged as a powerful platform for energy conversion devices. Researchers have demonstrated improved energy conversion efficiencies in photovoltaics and photocatalysis by combining materials with complementary properties, such as light absorption, charge separation, and transport. These novel heterostructures open up new opportunities for designing high-performance and multifunctional energy conversion devices.

Caroline^225^ have demonstrated that 2D van der Waals heterostructure based on mixed chalcogenides such as bitransition layers, have increased performance in supercapacitors due to lower layer stacking and better access for ions to increase the capacitance of the supercapacitors. Ma^226^ has developed a new 2D B_4_N_4_ monolayer that can store 8.2 wt% hydrogen at room temperature with desorption energy levels of 0.18–0.25 eV and the ability to reversibly release hydrogen at room temperature by optimizing the binding sites and using a light weight structure. Cheng^227^ has also fabricated a van der Waals heterostructure composed of BP/GeSe that exhibits a Type II band alignment with a band gap of 1.42 eV and exhibited ultra-fast electron and hole charge transfer times of 147 fs and 839 fs respectively; these heterostructures were also able to achieve 11.40% efficiency in converting photons into electrical current.

##### 5.3.5.4. MXene-based catalysts for fuel cells

MXene-based catalysts for fuel cells: fuel cells are promising clean energy technologies, and their commercialisation hinges on finding efficient and cost-effective catalysts. MXenes have shown remarkable catalytic activity for fuel cells' oxygen reduction and hydrogen evolution reactions. Recent breakthroughs in MXene-based catalysts have demonstrated enhanced performance and stability, reducing the reliance on expensive and rare platinum-based catalysts. These advancements can accelerate the adoption of fuel cells as a sustainable energy source for transportation and stationary power generation.^228–230^

Some improvements achieved using 2D materials:

• Energy density: energy density refers to the amount of energy that can be stored in a given volume or mass. 2D materials, such as graphene and MXenes, have high specific surface areas and can accommodate a large number of charge carriers, leading to increased energy storage capacity.^11^ The use of pseudocapacitive materials, like MXenes, in supercapacitors has significantly improved energy density due to their reversible redox reactions at the electrode–electrolyte interface.^231^

• Power density: power density represents the rate at which energy can be delivered or extracted from an energy storage device. 2D materials, with their high electrical conductivity, low resistance, and efficient charge transport properties, enable rapid charge–discharge rates and enhance power density in supercapacitors. Their fast kinetics and ability to handle high current densities result in quicker energy exchange, making them suitable for applications that require quick bursts of power.^232^

• Cycling stability: cycling stability refers to the ability of an energy storage device to maintain its performance over repeated charge–discharge cycles without significant degradation. 2D materials have demonstrated excellent cycling stability due to their strong chemical bonds and mechanical stability. Graphene, for instance, has shown negligible capacity loss over thousands of cycles in supercapacitors. The pseudocapacitive behavior of MXenes also contributes to their excellent cycling stability.

• Enhanced charge transfer kinetics: the high electrical conductivity of 2D materials, especially graphene, facilitates efficient charge transfer between the electrode and electrolyte, reducing internal resistance and enhancing energy and power densities.^233^

• Synergy with other materials: 2D materials can be integrated with other materials, such as metal oxides or conducting polymers, in hybrid supercapacitors. This combination of materials can result in synergistic effects, improving overall device performance and providing better energy density and cycling stability.

• Tunability: 2D materials offer tunable properties, allowing researchers to tailor their electronic structure and surface chemistry for specific energy storage applications. This tunability enables the optimization of energy and power densities to meet the requirements of various devices.^81^

The research of Asrar Alam provided an energy density in MXene-based asymmetric supercapacitor applications at 130.27 Wh kg^−1^ through pseudocapacitive redox reaction and the high surface area of FeZnS/MnZnS nanoparticle, thus significantly exceeding that of conventional capacitors. The study of Zhao^234^ reported a power density of 578 W kg^−1^ in flexible MXene pouch cells due to the high conductivity and reduced resistance pathways present within the two-dimensional (2-D) layered structure as compared to three-dimensional (3-D) materials. Zhang^221^ demonstrated a 90.3% retention of capacitance in MXene-rGO hybrid supercapacitor applications after 5000 cycles, primarily due to the mechanical stability of the MXene layer as well as the presence of strong covalent chemical bonds between the layers that prevent degradation. Nazarpour-Fard^235^ increased charge transfer through N-doped graphene/MXene electrode interfaces and maintained 90.4% of the capacitance retention up to 25 000 cycles by providing efficient electron pathways. A nanocomposite of WO_3_ nanoplates on ZnCo_2_O_4_ nanopetals combined with Ti_3_C_2_T_*x*_ MXene nanofibers (ZCO–WO_3_@MXNF) demonstrated enhanced structural stability, charge-transfer efficiency, and electron mobility, enabling a solid-state asymmetric supercapacitor with high energy density (28 Wh kg^−1^), power density (578 W kg^−1^), and 93% capacitance retention after 5000 cycles, proving its practical viability by powering LED lights.^236^

### 5.4. Future materials

#### 5.4.1. Single-atom catalysts on 2D supports

Single-atom catalysts (SACs) anchored on 2D materials like graphene, MXenes, and MoS_2_ maximize atom utilization and enable tunable redox sites, enhancing kinetics in batteries and supercapacitors. These are functionalized platforms, not standalone materials, where isolated metal atoms (*e.g.*, Fe, Co, Ni, Sn) create well-defined coordination environments.^237^

SACs on N-doped graphene, such as Co Sas/CoNC, facilitate uniform Li nucleation with strong binding energies (*e.g.*, −1.58 eV at H1 sites), suppressing dendrites and enabling 98.4% capacity retention over 340 cycles in Li|LFP cells.^238^ Fe/Co SAs on carbon supports deliver high capacities (*e.g.*, 14 777 mAh g^−1^) in Li–O_2_ batteries *via* efficient ORR/OER, outperforming Pt/C.^239^

On MXenes, Zn SAs enhance polyselenide anchoring per the Sabatier principle, optimizing Na–Se battery kinetics while avoiding overly strong binding that hinders charge–discharge. Co SAs on MoS_2_ basal planes *via* sulfur vacancies boost HER overpotentials to 17 mV, surpassing Pt/C.^240^

Challenges include atom migration under high currents/potentials. Future designs emphasize stable M–N–C or M–O–C coordination and wide-band-gap supports like h-BN, oxides, or nitrides to decouple sites from degradation, ensuring durability in oxidizing/reducing regimes.^241^

#### 5.4.2. Polyoxometalate-2D material hybrids

Polyoxometalates (POMs), redox-active metal-oxide clusters with tunable potentials, form molecular 2D hybrid electrodes *via* hydrogen bonding on graphene oxide (GO), MXenes, and RGO, enabling proton-coupled electron transfer (PCET) and dense H-bond networks for fast ion transport.^242,243^

These hybrids leverage POMs' multi-electron redox (“electron sponge” behavior) and 2D supports' conductivity, boosting pseudocapacitance; *e.g.*, asymmetric Mo/W POMs on GO/MXene deliver 384 F g^−1^ at 1 A g^−1^ with 91.7% retention after 2000 cycles, achieving 26 Wh kg^−1^ in HSCs. H-bonded POM gels and POMOFs enhance capacitance and lifetime *via* self-healing, as in PMo_12_/RGO with high energy/power density from synergistic ion transport.^244^

Challenges include oxidative instability and leaching in polar electrolytes. Future strategies: covalently/ionically lock POMs into hosts retaining H-bonds; couple with SACs or conductive 2D carbons for redox-mechanical stability.

#### 5.4.3. Rethinking MOFs, COFs, ternary dichalcogenides

MOFs and COFs offer ultrahigh surface areas (>7000 m^2^ g^−1^) and tunable porosity for ion accommodation, excelling in pseudocapacitive storage *via* linker/metal redox. However, poor intrinsic conductivity (<10^−8^ S cm^−1^), redox-inactive linkers, and framework collapse under high voltages limit direct electrode use.^245,246^

In contrast, 2D materials like graphene and MXenes provide superior electronic conductivity (>10^3^ S cm^−1^), mechanical flexibility, and electrochemical stability, enabling fast charge transport and cycle life >10 000 without additives.

Transition metal dichalcogenides (TMDs, *e.g.*, MoS_2_) and ternary sulfides/oxides (*e.g.*, NiCo_2_S_4_) deliver rich multi-electron redox and decent conductivity but suffer phase transformations, dissolution, and shuttle effects (*e.g.*, polysulfides in Li–S analogs).^247^

Scholarly positioning treats MOFs/COFs as sacrificial templates for heteroatom-doped carbons or oxides, yielding composites with retained porosity and band gaps; *e.g.*, ZIF-67-derived Co–N–C achieves 1200 mAh g^−1^ in Li–S with suppressed shuttling.^248^

TMDs/ternaries integrate into hierarchial heterostructures (*e.g.*, MoS_2_@TiO_2_, Cu_2_Mo_6_S_8_@carbon), where stable wide gap phases shield fragile actives, enhancing reversibility. This hybrid paradigm leverages 2D platforms' stability over standalone MOF/COF/TMD limitations.

#### 5.4.4. Large bandgap, oxidation-resistant materials

Ultrawide-bandgap (UWBG) materials like h-BN (5.9 eV), Ga_2_O_3_ (4.8 eV), AlN (6.2 eV), and diamond resist oxidation and corrosion at high potentials (>4 V), prioritizing stability over capacity in harsh electrolytes.^249^

h-BN hybrids excel as protective scaffolds; *e.g.*, Fe-BN/PANI electrodes deliver 721 F g^−1^ at 1 A g^−1^ with 81% retention after 10 000 cycles, leveraging h-BN's inertness for mechanical/electrochemical durability. BCN (borocarbonitride) variants enhance oxidation resistance beyond graphene in supercapacitors.^250^

Surface passivated MXenes (*e.g.*, polymer-coated Ti_3_C_2_T_*n*_) maintain capacitance (85% after 5000 cycles) by blocking nucleophilic attacks, enabling high-voltage operation.^251^

Oxide-protected MoS_2_ architectures, such as MoS_2_@TiO_2_ or GO-wrapped 3D MoS_2_, suppress dissolution and shuttling; 3D MoS_2_/GO retains 37% capacity (277 mAh g^−1^) after 3000 cycles at 5 A g^−1^ in Na-ion batteries.^252^

Wide-bandgap oxides (Ga_2_O_3_, AlN) integrate with 2D layers as electron/ion-blocking shells around SACs/TMDs, defining sharp potential windows and preventing parasitic reactions.^253^

Forward designs feature UWBG phases for stability encasing active sites (SACs, POMs, TMDs), shifting focus from capacity to long-term viability in next-gen devices. Key future design principles for advanced electrode materials emphasize decoupling redox activity from structural stability. This involves spatially separating highly active motifs like single-atom catalysts (SACs), polyoxometalates (POMs), and transition metal dichalcogenides (TMDs) from robust structural backbones composed of wide-band-gap oxides, nitrides, or BN like frameworks. Simultaneously, engineers must create multi-scale porosity with strong interfacial bonding either covalent or coordination-based to ensure H-bonded networks, MOF/COF derived carbons, and POM domains retain intimate contact and conductivity throughout prolonged cycling.

A core metric should target broad electrochemical stability windows, compressing high anodic and cathodic limits. Ultrawideband-gap (UWBG) and oxidation-resistant materials serve here not as primary capacity providers but as vital enablers, facilitating safe, durable operation at elevated voltages.

## 6. Challenges and future directions

In recent years, due to their unique properties, 2D materials have garnered significant attention as potential candidates for energy storage applications. However, their practical implementation faces various challenges and limitations. In this overview the current hurdles in utilising 2D materials for energy storage and delves into the ongoing research efforts and strategies aimed at overcoming these obstacles to unlock their full potential in revolutionizing energy storage technologies.

### 6.1. Challenges in utilizing 2D materials for energy storage

Scalability: one of the primary challenges is the scalable synthesis and production of high-quality 2D materials. Many 2D materials are still produced in small quantities in research laboratories, making it challenging to meet the demands of large-scale energy storage applications. Developing scalable synthesis methods and production processes is crucial for the widespread commercialization of 2D material-based energy storage devices.

Stability and degradation: some 2D materials can be susceptible to degradation over extended cycling, which can lead to a decrease in their electrochemical performance. The stability of 2D materials in various electrolyte environments and under different operating conditions needs to be thoroughly investigated and improved to ensure long-term performance and durability of energy storage devices.

Binder and electrode integration: integrating 2D materials into practical battery electrodes requires suitable binders and current collectors to maintain structural integrity and enhance electrical conductivity. Finding compatible binders that do not hinder ion transport while maintaining good adhesion to the electrode materials is essential for optimizing the overall electrode performance.

Interface engineering: the electrode–electrolyte interface plays a crucial role in energy storage performance. Improving the interaction between 2D materials and the electrolyte is essential to enhance ion diffusion rates and prevent unwanted side reactions, such as electrolyte decomposition or dendrite formation. Proper interface engineering is vital to achieving high efficiency and stability in 2D material-based energy storage devices.

Safety concerns: some 2D materials may exhibit high surface reactivity, leading to potential safety concerns, especially in lithium-ion batteries. Addressing safety issues, such as the risk of thermal runaway or electrode instability, is critical for the commercial adoption of 2D material-based energy storage technologies.

### 6.2. Potential solutions

Synthesis optimization: continuous efforts are needed to develop improved synthesis techniques that allow the scalable production of high-quality 2D materials. Methods like chemical vapor deposition and liquid exfoliation can be further optimized to achieve large-scale production while maintaining material quality and purity.

Interface modification: tailoring the surface chemistry of 2D materials through functionalization can improve their stability and interaction with electrolytes. Proper surface modification can enhance ion diffusion and suppress undesirable side reactions, leading to improved energy storage performance.

Composite and hybrid structures: integrating 2D materials into composite or hybrid electrode structures with other functional materials can enhance overall performance. Combining the unique properties of 2D materials with other materials like carbon nanotubes or metal oxides can synergistically improve energy storage properties.

Advanced characterization techniques: advanced characterization techniques, such as *in situ* microscopy and spectroscopy, can provide real-time insights into the electrochemical behavior of 2D materials during charge–discharge cycles. These techniques can help identify degradation mechanisms and guide the design of more stable materials.

Recycling and sustainability: developing sustainable and eco-friendly approaches for the synthesis, utilization, and recycling of 2D materials is essential. This includes exploring renewable energy sources and environmentally friendly chemical processes to minimize the environmental impact of energy storage technologies.

Safety testing and standardization: rigorous safety testing and standardization protocols are crucial to ensure the safe deployment of 2D material-based energy storage devices. Collaborative efforts among researchers, industry, and regulatory bodies are necessary to establish safety guidelines and ensure compliance with safety standards.

Ongoing research efforts in energy storage systems are increasingly focusing on the integration of 2D materials, presenting a promising avenue for advancements. One key strategy involves the development of hybrid structures, where 2D materials are combined with other nanomaterials or conductive additives. This approach seeks to capitalize on the synergies between different materials, leveraging their unique properties to address individual limitations and enhance both electrical conductivity and structural stability.

Atomic engineering emerges as another crucial facet in optimizing the performance of 2D materials. Tailoring the structure and composition at the atomic level through techniques like doping and alloying allows researchers to modify the inherent properties of these materials, ultimately improving their energy storage capabilities. Nanostructuring represents an innovative approach to tackle challenges associated with volume change during cycling. By creating nanostructured architectures from 2D materials, researchers aim to mitigate the mechanical stress induced by lithium intercalation. This nanoscale design facilitates the accommodation of strain and enhances overall mechanical robustness.

Interface engineering stands out as a key consideration in enhancing charge transfer efficiency and reducing resistance within energy storage devices. Surface functionalization and the use of suitable electrolytes are explored to optimize the interface between 2D materials and electrodes, consequently improving the overall energy storage performance. Advanced synthesis techniques play a vital role in the scalability and cost-effectiveness of incorporating 2D materials. Ongoing efforts involve the exploration of methods such as chemical vapor deposition and liquid exfoliation, aiming to develop high-quality 2D materials for widespread application in energy storage systems.

Safety concerns associated with 2D materials in energy storage devices are also a focal point of research. Investigating and implementing protective coatings, stable electrolytes, and effective thermal management strategies are imperative for enhancing the safety profile of these materials. In the realm of computational science, researchers rely on sophisticated modeling techniques to predict the behavior of 2D materials during energy storage processes. These computational simulations aid in identifying promising candidates, guiding the optimization of their performance for practical applications Also researchers are exploring novel device architectures and designs to maximize the benefits of 2D materials in energy storage. Flexible and 3D configurations are being investigated for their potential to offer improved efficiency and energy density, paving the way for the next generation of energy storage systems within the broader domain of polymer science and engineering.

### 6.3. Emerging trends

The field of 2D materials for energy storage is rapidly evolving, and several emerging trends and future prospects are shaping research and development direction. Some of the key trends and prospects include:

Novel 2D materials discovery: researchers are continually exploring and synthesizing new 2D materials with unique properties that can further enhance energy storage performance. The discovery of novel materials, beyond the well-known graphene and MXenes, opens up new opportunities for designing advanced energy storage devices with superior properties.

2D material composites: hybrid structures that combine 2D materials with other functional materials, such as metal oxides, conducting polymers, or nanomaterials, are gaining interest. These composites exhibit synergistic effects, combining the strengths of each component to achieve enhanced energy storage performance.

Heterostructures and stacking configurations: as researchers delve into stacking multiple layers of different 2D materials, heterostructures with tailored electronic properties and charge transport characteristics are being explored. These heterostructures hold promise for fine-tuning energy storage behavior and overcoming limitations associated with individual 2D materials.

Beyond lithium-ion batteries: 2D materials are also being investigated for use in alternative energy storage technologies beyond conventional lithium-ion batteries. For instance, they hold potential for supercapacitors, sodium-ion batteries, potassium-ion batteries, and even metal–air batteries, expanding the scope of applications in different energy storage systems.

Flexible and wearable energy storage: the exceptional mechanical flexibility and thin nature of 2D materials make them ideal candidates for flexible and wearable energy storage devices. The prospect of integrating 2D materials into clothing, wearable electronics, and flexible electronic devices is gaining traction.

Scalability and commercialization: advancements in large-scale production and processing techniques are critical for commercializing energy storage devices based on 2D materials. As scalable manufacturing processes are developed, the market penetration of 2D material-based energy storage technologies is expected to increase.

Integration with other technologies: the integration of 2D material-based energy storage with other emerging technologies, such as Internet of Things (IoT), electric vehicles, and renewable energy systems, can revolutionize energy storage solutions for a sustainable future.

Safety and environmental impact: research is ongoing to ensure the safety and environmental impact of 2D material-based energy storage technologies. Understanding the potential toxicity and recycling of these materials is essential for their widespread adoption and sustainable implementation.

Computational modeling and machine learning: computational approaches, such as density functional theory and machine learning algorithms, are being employed to accelerate the discovery and optimization of 2D materials for energy storage applications.

Cross-disciplinary collaborations: the field of 2D materials for energy storage benefits from cross-disciplinary collaborations involving materials science, chemistry, physics, engineering, and electrochemistry. Such collaborations foster innovation and accelerate progress in the development of cutting-edge energy storage technologies.

The future of 2D materials for energy storage holds immense promise, with ongoing research efforts focusing on discovering new materials, optimizing device design, and addressing scalability and safety concerns. As these emerging trends unfold, 2D materials are likely to play a significant role in shaping the landscape of next-generation energy storage technologies.

## 7. Outlook & future challenges

Although 2D materials have made significant strides toward developing new forms of energy storage, many important technical hurdles must be overcome before they may be commercially viable on an enormous scale. Thus, it is not enough for future research to focus solely on increasing the internal material properties of 2D materials; researchers must also develop solutions to the practical problems associated with processing, stability, safety and repeatability.

A major hurdle is the chemical stability and long-term reliability of MXenes due to susceptibility to oxidation upon exposure to air, during storage, processing and cycling. A small amount of oxidation can cause substantial reductions in both conductivity and electrochemical activity. Therefore, it will be essential to develop protective surface coatings using antioxidants, to implement methods of solid-state storage or to identify scalable means of encapsulating MXene materials so that they retain their performance characteristics in actual operating conditions. In addition, graphene-based materials frequently undergo “restacking,” which reduces the available surface area and ion-accessible sites. To prevent this restacking, researchers will need to develop nanostructured architectures for graphene-based materials, introduce spacers between layers or create three-dimensional porous frameworks of graphene in order to maintain the structural openness and facilitate optimal charge-transport pathways through the material.

Semiconductor-phase MoS_2_ has a limited rate capability and ability to perform high power applications due to its relatively low intrinsic electrical conductivity. The direction of future research includes controlling the phase composition (*i.e.*, 2H → 1T transition) of semiconductor-phase MoS_2_ and modulating defects within the crystal structure of MoS_2_. Additionally, researchers should consider forming heterostructures by combining MoS_2_ with conductive scaffolding (graphene, MXene, CNTs) to increase conductivity while maintaining structural integrity. Achieving mechanical robustness, high mass loading and scalable fabrication of binder-free electrodes remains a primary challenge for both flexible and thick-film configurations.

In addition to material-related challenges, the field faces broader manufacturing and sustainability-related concerns. Most of the synthesis processes currently used for 2D materials such as HF-based MXene etching and high temperature chemical vapor deposition, for example are either extremely energy intensive or dangerous and not feasible for large-scale manufacturing. It is essential to develop green, inexpensive and scalable synthesis techniques that meet sustainability criteria for use in manufacturing 2D materials. Furthermore, environmental and safety factors, including handling of toxic chemicals, long-term disposal of nanomaterials, and life cycle assessment, must be included in future research to ensure safe and environmentally friendly implementation of 2D materials.

The field is currently in great need of standardized electrochemical test and reporting protocols to compare studies since there is little standardization regarding how electrodes are prepared, mass loaded, what electrolytes are used, and what test procedures are employed. Standardized performance metrics for capacitance, capacity, rate testing, coulombic efficiency and cycling stability would greatly improve the reproducibility of results across studies and ultimately accelerate the selection of appropriate materials for various applications.

Therefore, the road ahead will require complementary advancements in materials chemistry, device engineering, manufacturing and test standards to realize the potential of 2D materials and create scalable, safe and high-performance energy storage technologies that meet global demand.

## 8. Conclusion

In a nutshell, this review has highlighted the remarkable progress and significant advancements made in utilising 2D materials for energy storage applications. The unique properties and surface reactivity of 2D materials have enabled diverse energy storage mechanisms, including intercalation, adsorption, pseudo-capacitance, and conversion reactions. These mechanisms offer a wide range of options for designing advanced energy storage devices with improved energy density, power density, and cycling stability.

One of the key findings of this review is the exceptional performance of 2D materials, such as MXenes and graphene, in supercapacitors and capacitive energy storage devices. Their high surface area, fast ion diffusion, and pseudocapacitive behaviour have led to ultrahigh energy densities and rapid charge–discharge rates. Moreover, advancements in electrode materials, electrolytes, and overall battery performance have propelled the development of high-performance lithium-ion batteries and beyond.

The significance of 2D materials in energy storage lies in their potential to revolutionise the energy landscape. Their tunable properties, scalability, and environmental sustainability offer viable solutions for the growing global demand for efficient and clean energy storage technologies. The breakthroughs in perovskite-like 2D materials for solar cells, hydrogen storage on 2D materials, and MXene-based catalysts for fuel cells hold promise for a sustainable energy future.

The potential impact of future developments in this area is profound. As research continues to unveil the intrinsic properties of 2D materials and innovative techniques to harness their full potential, we can expect even greater advancements in energy storage technologies. With the possibility of high-capacity batteries, efficient fuel cells, and enhanced solar cells, 2D materials are poised to play a crucial role in transitioning to a sustainable energy landscape.

The versatility and multifunctionality of 2D materials position them as a fundamental element in energy storage. As we work to address global energy issues, ongoing research and the use of 2D materials are crucial for developing innovative energy storage solutions that support a cleaner, greener, and more sustainable future.

## Author contributions

Sebin Kariachan: data curation, formal analysis, investigation, methodology, validation, writing – original draft; Jibin K. P.: conceptualization, formal analysis, investigation, methodology, validation, supervision, writing – original draft, writing – review and editing; Pravitha V., Joshin Shibu, Sisanth K. S., Sanu Mathew Simon and Prajitha V: investigation, methodology, writing – review and editing; Hitoshi Kasai: writing – review and editing; Sabu Thomas: conceptualization, methodology, project administration, resources, supervision, validation, writing – review and editing: Kohei Okubo and Kouki Oka: resources, supervision, validation, writing – review and editing.

## Conflicts of interest

The authors declare no conflicts of interest.

## Data Availability

No data was used for the research described in the article.
